# Identification of the Recombinant *Plasmodium vivax* Surface-Related Antigen as a Possible Immune Evasion Factor Against Human Splenic Fibroblasts by Targeting ITGB1

**DOI:** 10.3389/fcell.2021.764109

**Published:** 2021-12-06

**Authors:** Haitian Fu, Jiachen Lu, Xinxin Zhang, Bo Wang, Yifan Sun, Yao Lei, Feihu Shen, Kokouvi Kassegne, Eun-Taek Han, Yang Cheng

**Affiliations:** ^1^ Affiliated Hospital of Jiangnan University, Wuxi, China; ^2^ Laboratory of Pathogen Infection and Immunity, Department of Public Health and Preventive Medicine, Wuxi School of Medicine, Jiangnan University, Wuxi, China; ^3^ Yantai Center for Disease Control and Prevention, Yantai, China; ^4^ Department of Clinical Laboratory, The First Affiliated Hospital of Anhui Medical University, Hefei, China; ^5^ Chinese Center for Tropical Diseases Research, School of Global Health, Shanghai Jiao Tong University School of Medicine, Shanghai, China; ^6^ Department of Medical Environmental Biology and Tropical Medicine, School of Medicine, Kangwon National University, Chuncheon, South Korea

**Keywords:** *Plasmodium vivax*, immune evasion, human splenic fibroblasts, *Plasmodium vivax* surface-related antigen, ITGB1

## Abstract

*Plasmodium vivax*–infected erythrocytes can enter the spleen and evade spleen clearance to establish chronic infections. However, the mechanism underlying *P. vivax* immune evasion in the spleen is still unclear. Human splenic fibroblasts (HSF), also known as barrier cells, play an essential role in the immune function of spleen. A hypothesis holds that *P. vivax*—infected erythrocytes induce spleen structural remodeling to form barrier cells. Subsequently, these infected erythrocytes can selectively cytoadhere to these barrier cells to escape spleen clearance. In this work, we found that *P. vivax* surface-related antigen (PvSRA; PlasmoDB ID: PVX_084970), an exported protein on infected erythrocyte membrane, could bind with HSF. Considering the above hypothesis, we speculated that PvSRA might be involved in *P. vivax* immune evasion by changing HSF cell performance. To investigate this speculation, RNA sequencing, protein microarray, and bioinformatics analysis technologies were applied, and *in vitro* validations were further performed. The results showed that the recombinant PvSRA attracted HSF migration and interacted with HSF by targeting integrin β1 (ITGB1) along with changes in HSF cell performance, such as focal adhesion, extracellular matrix, actin cytoskeleton, and cell cycle. This study indicated that PvSRA might indeed participate in the immune evasion of *P. vivax* in the spleen by changing HSF function through PvSRA–ITGB1 axis.

## 1 Introduction

Globally, the World Health Organization estimates that, in 2019, 229 million clinical cases of malaria occurred and 409,000 people died of malaria (Varo et al., 2020; World Health Organization, 2020). Although tertian ague caused by *Plasmodium vivax* was once thought to be benign when compared with falciparum malaria induced by *Plasmodium falciparum*, recent reports found that, in many co-endemic malarious areas, the most commonly transmitted malaria parasite after *P. falciparum* parasite is *P. vivax*, and about a quarter of the cases in 2019 were due to *P. vivax*, mainly in Afghanistan and Pakistan (World Health Organization, 2020). *P. vivax* parasitemia is associated with substantial morbidity including a cumulative risk of severe anemia, hypohepatia, acute lung injury, hypersplenism, splenomegaly, and acute renal failure, whereas its pathogenesis is still unclear (Douglas et al., 2013; Commons et al., 2019). Among these clinical symptoms, splenomegaly is one of the most common features of malaria, and splenic rupture is more prevalent among infections caused by *P. vivax* compared with other species (Elizalde-Torrent et al., 2018). It has been found that plasmodium parasite evades clearance of host immune system in the erythrocytic stage to cause malaria symptoms (Bassat and Alonso, 2011; Douglas et al., 2013; Im et al., 2017; Commons et al., 2019). Clinical study has found that a large number of *P. vivax*—infected erythrocytes (Pv-iRBCs) were located in the red pulp of spleen, indicating that *P. vivax* could evade the immune clearance of spleen (Machado Siqueira et al., 2012). However, the precise mechanism is still unknown.

During the research of *P. falciparum*, it was found that the variant surface antigens of *P. falciparum* play an essential role in its immune evasion. For instance, as a member of the variant surface antigens, *P. falciparum* erythrocyte membrane protein-1 (PfEMP1) located at the knob on the infected erythrocyte surface is a ligand of several hose cell receptors, such as platelet endothelial cell adhesion molecule 1 (CD31) on the endotheliocyte surface, complement receptor type 1 on the erythrocyte surface, and intercellular adhesion molecule-1 (ICAM-1) (Chen et al., 2000). Therefore, *P. falciparum*—infected erythrocytes (Pf-iRBCs) could bind to uninfected erythrocytes to form rosettes and further bind to host vascular adhesins. Besides, increased rigidity and lowly deformability of Pf-iRBC further allow them to evade the host immune attack by adhering to the blood vessel endothelium instead of entering the spleen (Chen et al., 2000; Turner et al., 2013). However, Pv-iRBC cytoadhesion levels are 10-fold lower than those observed for Pf-iRBC, and Pv-iRBCs are still deformable, allowing them enter the spleen (Bozdech et al., 2008; Carvalho et al., 2010), indicating that the immune evasion mechanism of *P. vivax* might be different from that of *P. falciparum*.

Human splenic fibroblasts (HSF), also named as barrier cells, are deployed as diverse barriers in the splenic pulp. When the spleen is attacked by pathogens, these cells rapidly and greatly increase in number to form splenic filtration beds that are important in the increased capacities of spleen in clearance, phagocytosis, and immunological reactivity (Weiss, 1991). A non-lethal murine model of plasmodium, *P. yoelii* strain 17X, can induce spleen-resident fibroblasts to form physical barrier cells, causing the “open” circulation of the spleen to suddenly and temporarily change into a “closed” circulation (Weiss, 1989). Because of similarities between *P. yoelii* strain 17X infections in BALB/c mice and *P. vivax* infections, a hypothesis holds that Pv-iRBCs induce spleen structural remodeling to form barrier cells and, subsequently, selectively undergo cytoadherence to escape spleen clearance (del Portillo et al., 2004). However, this hypothesis has not been proved.

In present work, we found that *P. vivax* surface-related antigen (PvSRA), an exported antigen from *P. vivax* to the surface of the infected erythrocyte membrane, could bind with HSF. Considering the important role of surface antigen in the erythrocytic stage (Chen et al., 2000), together with the above hypothesis, we suspected that PvSRA might be involved in *P. vivax* immune evasion in the spleen. To investigate this speculation, RNA sequencing, protein microarray, and bioinformatics analysis technologies were applied to predict the potential function of PvSRA, and *in vitro* validations were further performed. The results indicated that recombinant PvSRA attracted HSF migration and interacted with HSF by targeting integrin β1 (ITGB1) along with some changes in HSF cell performance. This study indicated that PvSRA might indeed participate in the immune evasion of *P. vivax* in the spleen by changing HSF function through PvSRA–ITGB1 axis. This finding will cast new light on the research of the pathogenesis of *P. vivax*.

## 2 Materials and Methods

### 2.1 Reagents and Equipment

Anti-green fluorescent protein (GFP) (ab183734), anti-six-histidine tags (6×His) (ab9108), anti-ITGB1 (ab134179), anti-matrix metalloproteinase-1 (MMP-1) (ab134184), anti-carboxypeptidase A3 (CPA3) (ab251685), anti-tissue factor pathway inhibitor 2 (TFPI2) (ab186747), anti-myosin heavy chain 3 (MYH3) (ab124205), anti-focal adhesion kinase (FAK) (ab40794), and anti-glyceraldehyde-3-phosphate dehydrogenase (GAPDH) (ab181602) were obtained from Abcam, Inc. (Cambridge, United States). Anti-phospho-mitogen-activated protein kinase family (MAPK) (9926T) and anti-MAPK family (9910T) were obtained from Cell Signaling Technology, Inc. (Beverly, United States). Secondary antibodies, including horseradish peroxidase (HRP)-conjugated anti-rabbit antibody (A0208), HRP-conjugated anti-mouse antibody (A0216), and HRP-conjugated anti-human antibody (A0201). Enhanced chemiluminescence kit, CCK8 test kit, RIPA lysis buffer and NP40 lysis buffer were obtained from Beyotime Biotechnology (Shanghai, China). Human cartilage oligomeric matrix protein (COMP) ELISA Kit (JL11535) was purchased from JiangLai Biotechnology (Shanghai, China). FxCycle PI/PNase staining solution, Lipofectamine 3000, anti-human ITGB1 antibody (TS2/16) for functional assay, Endotoxin Removing Gel, LAL Chromogenic Endotoxin Quantitation Kit, APC-anti-integrin subunit alpha 8 (ITGA8) (481709), Alexa Fluor 546–conjugated goat anti-rabbit (A11035), and 4′,6-diamidino-2-phenylindole were obtained from Thermo Fisher Scientific, Inc. (Shanghai, China). Anti-GFP mAb magnetic agarose was obtained from MBL Beijing Biotech Co., Ltd. (Beijing, China). RNA isolation reagent was obtained from Yishan, Inc. (Suzhou, China). Fibroblast medium was purchased from ScienCell Research Laboratories (Carlsbad, United States). CY3 MONO 5-PACK was obtained from Sigma-Aldrich Corporation (MO, United States). APC–anti–His-tag (362605), APC–anti–programmed death ligand-1 (PD-L1) (329707), and PE–anti-ITGB1 (303003) were obtained from BioLegend (San Diego, United States) for FACS analysis.

HuProt^™^ version 3.1 array was provided by CDI Laboratories, Inc. (Mayaguez, United States). Luxscan^™^ 10Κ-A microarray scanner was provided by CapitalBio Corporation (Beijing, China). Inverted fluorescence microscope was obtained from Nikon Corporation (Tokyo, Japan). Multiskan GO microplate reader was obtained from Thermo Fisher Scientific, Inc. (Shanghai, China). Confocal microscope was provided by Olympus Optical Co., Ltd. (FV200, Tokyo, Japan). Flow cytometer was obtained from BD Biosciences (C6, Shanghai, China).

### 2.2 Cells and Bacterial Strain

*Escherichia coli* BL21 strain was obtained from TransGen Biotech Co., Ltd. (Beijing, China). pET28a expression vector was obtained from YouLong Biotech Co., Ltd. (Shanghai, China). HSF were purchased from ScienCell Research Laboratories (5530, Carlsbad, United States).

### 2.3 Preparation of Recombinant PvSRA

**FIGURE 1:**
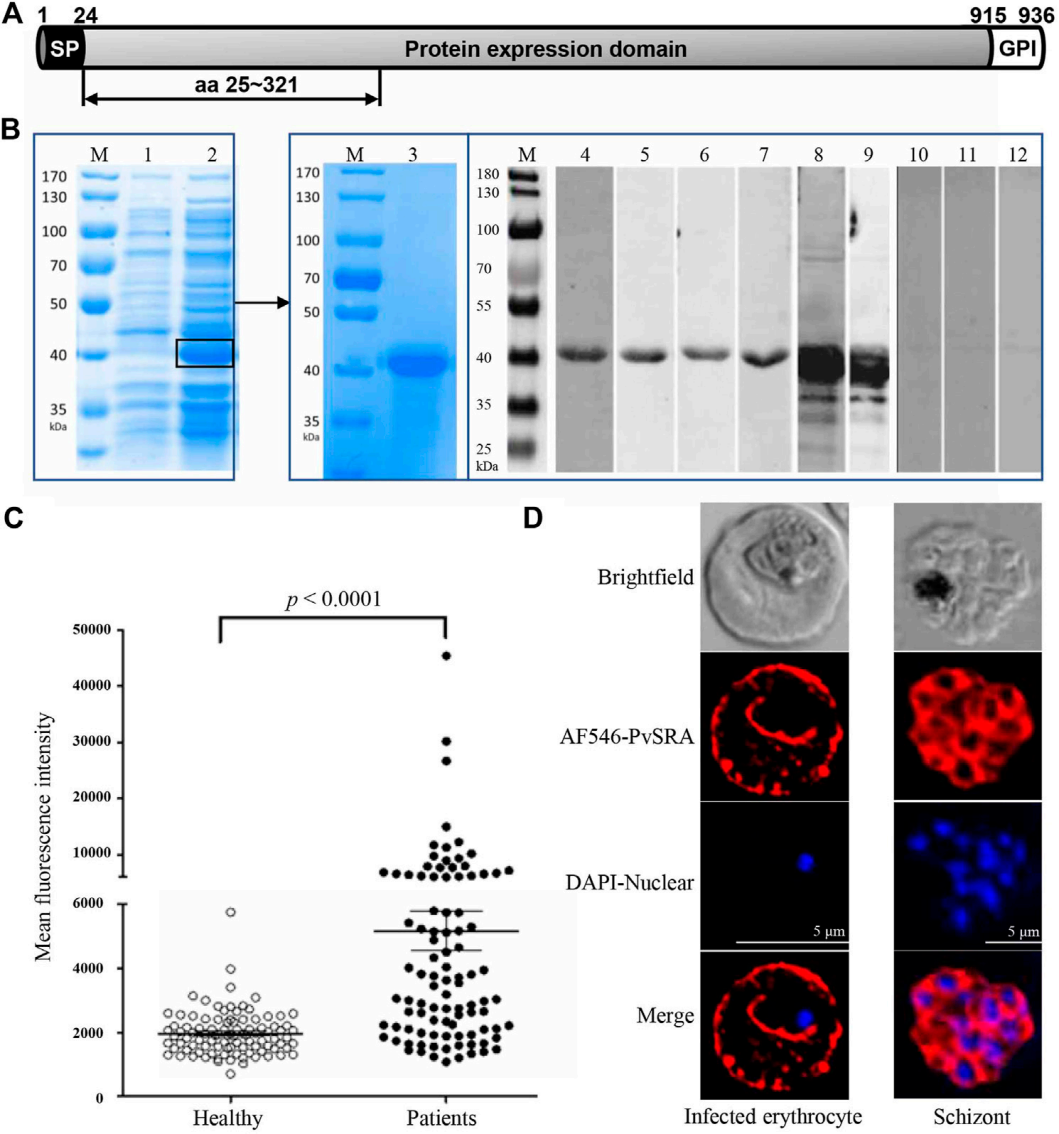
Expression of recombinant PvSRA and location of PvSRA on infected erythrocytes. **(A)** Schematic diagram of gene sequence of PvSRA. PvSRA consists of a signal peptide (SP) [amino acids (aa) 1–24], a protein expression domain (aa 25–914), and a GPI-anchored domain (aa 915–936). The functional region of PvSRA with a total of 297 aa (aa 25–321) was expressed in *E. coli* BL21 in present work. **(B)** Expression of PvSRA functional region protein. The recombinant PvSRA protein was detected by anti–6×His-tag antibody and specific sera in WB analysis. M, Protein marker; lane 1, *E. coli* BL21 strain containing pET28a-PvSRA; lane 2, BL21 cell containing pET28a-PvSRA with 0.5 mM IPTG induction; lane 3, SDS-PAGE analyses for the purified PvSRA; lane 4, WB analyses for the purified PvSRA with anti-6×His antibody; lanes 5–7, sera prepared from three *P. vivax*–infected patients; lane 8, specific sera prepared from rabbit; lane 9, specific sera prepared from mouse; lane 10, sera from healthy individual; lane 11, sera from normal rabbit; and lane 12, sera from normal mouse. **(C)** Comparison of IgG antibody responses to recombinant PvSRA proteins. Sera samples from *P. vivax*–infected patients (*n* = 96) and healthy individuals (*n* = 96) were used for measurement of antibody responses by protein array. The bar indicates mean value and 95% confidence interval. MFI, mean fluorescence intensity. **(D)** Localization of PvSRA protein in the erythrocytic and mature schizont stages of *P. vivax* parasites by using the specific rabbit antisera against PvSRA and Alexa Fluor 546–conjugated goat anti-rabbit IgG. Nuclei are visualized with DAPI in merged images. Bars represent 5 μm.

The full-length gene sequence of PvSRA (PlasmoDB ID: PVX_084970) was obtained from GenBank (https://plasmodb.org/plasmo/), and it was predicted as an extracellular protein without transmembrane domain (http://www.cbs.dtu.dk/services/TMHMM/). PvSRA consists of a signal peptide (SP) [amino acids (aa) 1 to 24], a protein expression domain (aa 25–914), and a GPI-anchored domain (aa 915–936). The full protein expression domain with a total of 890 aa of PvSRA was expressed hardly in *E. coli* BL21. Then, the functional region of PvSRA was identified by using the consensus prediction method available at the Immune Epitope Database and Analysis Resource (http://www.iedb.org/), which is contained in the first one-third of the full protein expression domain (Figure 1A). Hence, this fragment with a total of 297 aa (aa 25–321), with 6×His added at the C-terminal end, was expressed by using the pET28a expression vector in *E. coli* BL21 strain in LB medium at 37°C and 200 rpm and induced with 0.5 mM isopropyl-β-d-thiogalactoside for another 5 h when OD600 value was 0.6–0.8. Crude protein was purified through Ni-chelating affinity chromatography by YouLong Biotech (Wuxi, China). PvSRA expression was verified by SDS-PAGE and Western blot (WB) analysis by using anti-6×His antibody. Specific sera prepared from immunized rabbit and mouse was also used to verify the expression of PvSRA. Moreover, sera collected from randomly selected *P. vivax*—infected patients were used to test the expression level of PvSRA. Sera from normal mice and rabbits, as well as those from healthy individuals, were used as negative controls.

To exclude the effects of endotoxin in the PvSRA, the endotoxin was removed with Endotoxin Removing Gel, and the residues were determined with a LAL Chromogenic Endotoxin Quantitation Kit. After the treatment, the endotoxin was very low (<0.01 ng/ml) in PvSRA at a concentration of 100 μg/ml.

### 2.4 Preparation of Specific Sera Against PvSRA

The specific sera against PvSRA were prepared as follows. The rabbit group received 250 μg of purified PvSRA in 2 ml of saline emulsified with an equal volume of adjuvant through intramuscular injection, whereas the mouse group received 20 μg of purified PvSRA in 0.1 ml of saline emulsified with an equal volume of Freund’s adjuvant through intraperitoneal injection. For the first immunization, Freund’s complete adjuvant was used, followed by further immunizations 4 and 6 weeks later with Freund’s incomplete adjuvant, and the antisera were collected 2 weeks after the last immunization.

### 2.5 Protein Array Analysis

#### 2.5.1 Human Plasma Sample Collection

In this experiment, 5 ml of positive serum samples were collected from 96 patients who were confirmed positive for *P. vivax* infection by microscopy (0.123% mean parasitemia; range 0.034–0.501%) at the local health centers and clinics in Gangwon Province in malaria-endemic areas of the Republic of Korea, and RT-PCR was also applied to avoid inclusion of a sample with coinfection of *P. falciparum* and *P. vivax*. Among the patients, 15 were aged 3–19 years, 24 were aged 19–25 years, 21 were aged 26–39 years, 18 were aged 40–49 years, and 18 were aged 50–79 years. Ninety-six plasma samples taken from healthy personnel who were confirmed negative for *P. vivax* malaria by microscopy and had no malaria episodes in the past were used as negative controls. Among these healthy volunteers, 21 were aged 15–25 years, 43 were aged 26–39 years, 18 were aged 40–49 years, and 14 were aged 50–65 years.

#### 2.5.2 Protein Microarray

All the sera were tested against the recombinant PvSRA protein using protein array analysis according to our previous studies with slight modification (Chen J.-H. et al., 2010; Lu et al., 2014). To determine the use concentration of PvSRA in present experiment, the correlation between duplicate spots of the protein arrays and antibody reactivity of different concentrations of the recombinant proteins was first analyzed by using Origin software (OriginLab Corp., Northampton, MA). In brief, PvSRA in PBS with different concentration was spotted in duplicates onto modified glass slides, incubated for 2 h at 37°C, and blocked with 5% BSA in PBS/Tween 20 (PBST) buffer for 1 h at 37°C. Anti–His-tag antibody solution (50 μg/ml) was added and incubated for 1 h, then Alexa Fluor 546–conjugated goat anti-rabbit (50 μg/ml) was added and incubated for another 1 h, and the mean fluorescence intensity (MFI) was measured by using a scanner (Supplementary Figure 1A). The correlation between duplicate spots of the protein arrays and antibody reactivity of different concentrations of the recombinant proteins was analyzed using Origin software. A correlation coefficient of 0.998 between fluorescence intensities and protein concentrations, and the antigen-antibody reaction was stable when the concentration was reached 50 ng/μl (Supplementary Figure 1B). Therefore, this value was selected as the sample concentration on protein microarray.

Recombinant PvSRA protein in PBS (0.9 μl/spot, 50 ng/μl) was spotted in duplicates onto Ni^2+^ chelated surface slide and incubated for 2 h, blocked with 5% BSA in PBST buffer for 1 h at 37°C, loaded with sera from patients and healthy controls (1:200, 1 μl/spot), and incubated for 1 h at 37°C. Wells were then stained with Alexa Fluor 546 goat anti-human IgG (10 ng/μl, 1 μl/spot) in PBST buffer (1% BSA) for 1 h at 37°C and scanned in a scanner. PvMSP1-19 was taken as positive control. For analysis, the positive cutoff value was calculated as the MFI value of the negative controls plus two standard deviations. Mann-Whitney *U* test was performed to compare differences in MFI between groups.

### 2.6 Indirect Immunofluorescence Assay

Indirect immunofluorescence assay was used to detect PvSRA localization in *P. vivax* erythrocytic-stage and mature schizont-stage parasites by confocal microscopy. In brief, mature intraerythrocytic-stage and mature schizont-stage *P. vivax* parasites collected from a malaria patient in Thailand were fixed with ice-cold acetone for 3 min on slides and then dried. After blocking with 5% nonfat milk in PBS at 37°C for 30 min, the slides were incubated with rabbit anti-PvSRA serum (1:100) at 37°C for 1 h and then subjected to fluorescence staining with Alexa Fluor 488–conjugated goat anti-rabbit. 4′,6-diamidino-2-phenylindole was used for nuclear staining at 37°C for 30 min. The fluorescence of the slides was visualized under oil immersion by a confocal laser scanning microscope. Images were captured with the FV10-ASW3.0 Viewer software.

### 2.7 HSF Cultures

HSF are characterized by their spindle morphology and immunofluorescence with antibodies specific to fibronectin, and its identification was provided by ScienCell Research Laboratories. HSF are guaranteed to further expand for 15 population doublings under their recommended culture method. In present work, the cell culture method and the passage number for using were also according to their instruction strictly. Briefly, HSF was cultured in accordance with the instructions of the provider. Briefly, primary cells were cultured in poly-l-lysine-coated culture vessels (2 μg/cm^2^) in a 37°C and 5% CO_2_ incubator. The fibroblast medium was changed every 3 days until the culture reached approximately 70% confluent. Once the culture reaches 70% confluency, the medium was changed every other day until the culture is approximately 90% confluent. Subculture was performed with trypsin/EDTA solution (0.05%) when the culture reaches 95% confluency.

### 2.8 Binding Analysis Between PvSRA and HSF

Binding between PvSRA and HSF was determined by flow cytometry and WB analysis. In brief, HSF were suspended in the medium alone or in a medium containing protein sample (50 μg/ml) at a density of 2 × 10^5^ cells per well in six-well flat-bottomed tissue culture plates. The plate was incubated at room temperature for 2 h with slight shaking. For FACS analysis, the cells were collected, washed with ice-cold PBS, and incubated with APC–anti–6×His-tag for 30 min at 4°C. Finally, the cells were analyzed by FACScan, and the binding was determined by FlowJo v10. For WB analysis, the collected cells were first lysed with RIPA lysis buffer, followed by SDS-PAGE and WB analysis with anti-6×His antibody. Besides, immunofluorescence was further applied to observe the binding between PvSRA and HSF. Simply, HSF were seeded in 24-well flat-bottomed tissue culture plates at a density of 2 × 10^4^ cells per well. After incubated with PvSRA (50 μg/ml) in complete culture medium at room temperature for 2 h, the cells were washed with cold PBS and fixed with 3.7% methanol-free formaldehyde solution in PBS for 15 min at room temperature. Then, the cells were blocked with 5% bovine serum albumin for 10 min, followed by incubation staining with FITC–anti–6×His-tag overnight at 4°C. After removing the residual staining reagents, binding between PvSRA and HSF was observed and recorded by inverted fluorescence microscope.

### 2.9 Transcriptome Analysis

To predict the effect of PvSRA on HSF, transcriptome analysis, also named RNA-seq technology, was applied in this work. After HSF were cultured with PvSRA at 50 μg/ml for 48 h, total RNA was extracted using RNA isolation reagent. Quantification, qualification, library preparation, and subsequent RNA-seq of RNA samples were conducted by Novogene Co., Ltd. (Tianjing, China). Differential expression analysis was performed using the edgeR R package (version 3.18.1). *p* values were adjusted using the Benjamini and Hochberg method. An adjusted *p* value (padj) less than 0.05 and an absolute fold change of 2 were set as thresholds for a significantly differential expression.

Gene Ontology (GO) enrichment is a technique for interpreting gene sets by making use of the GO system of classification, in which genes are assigned to a set of predefined bins depending on their functional characteristics (http://geneontology.org/). GO enrichment analysis of DEGs was implemented by using clusterProfiler R package, in which the gene length bias was corrected. GO terms with padj less than 0.05 were considered significantly enriched. Kyoto Encyclopedia of Genes and Genomes (KEGG) is a database resource for understanding high-level functions and utilities of biological systems, such as cell, organism, and ecosystem, from molecular-level information, especially large-scale molecular datasets generated by genome sequencing and other high-throughput experimental technologies (http://www.genome.jp/kegg/). clusterProfiler R package was used to test statistical enrichment of DEGs in KEGG pathways. KEGG pathways with *p* value less than 0.05 were considered significantly enriched.

### 2.10 Probe PvSRA Substrates on the Human Proteome Microarray

High-throughput protein microarray was used to track interactions between PvSRA and human proteome, and this experiment was conducted by Bochong Biotechnology Co., Ltd. (Guangzhou, China). Briefly, PvSRA labeled with CY3 MONO 5-PACK was added to the HuProt^™^ version 3.1 array that contains >20,000 unique proteins, representing 80% of the human proteome, whereas BSA labeled with CY3 was taken as negative control. After incubation overnight in the dark at 4°C, the array was blocked with 5% BSA in PBST for 1 h in the dark at room temperature, then washed with PBST, and air-dried. The reaction between PvSRA and the immobilized protein emits a fluorescent signal, which was read by a Luxscan^™^ 10Κ-A microarray scanner. To acquire the candidate list of PvSRA binding proteins, the signal-to-noise ratios (SNRs) were calculated for all the spots and then averaged from two duplicated spots. The cutoff was set as SNR (+) > 2 to determine the final positive hits.

The protein-protein interaction (PPI) network graph between the positive PvSRA substrates was drawn by using the STRING database (https://string-db.org/), and the degree of interaction was calculated by Cytoscape software. GO enrichment analysis was applied to these positive candidates from the following three aspects: cellular component (CC), molecular function (MF), and biological process (BP). The cutoff was set as *p* value less than 0.05 and fold enrichment index greater than 1. KEGG pathway enrichment analysis was also applied, and the cutoff was set as *p* value less than 0.05 and fold enrichment index greater than 1.

### 2.11 Co-Immunoprecipitation

Co-immunoprecipitation testing was applied to verify interaction between PvSRA and ITGB1 in HEK293 cells. Given that ITGB1 is expressed by HEK293, 4 μg of pEGFP-C1-PvRSA was transfected into 2 × 10^6^ cells with Lipofectamine 3000, alongside with pEGFP-C1 as negative control. The cells were cultured for 48 h and then collected and lysed with 400 μl of cold NP-40 lysis buffer. The lysate was collected and mixed with 30 μl of anti-GFP mAb magnetic bead agarose. Thereafter, the mixture was incubated with gentle agitation overnight at 4°C, and the supernatant was removed by using a magnetic rack. After the magnetic beads were washed with 1 ml of cold lysis buffer for five times, 50 μl of SDS-PAGE loading buffer was added and boiled for 3 min. Proteins were separated by SDS-PAGE and analyzed by WB analysis. Anti-GFP and anti-ITGB1 antibodies were used to reveal PvSRA and ITGB1 proteins, respectively. Detection was carried out by using anti-rabbit IgG HRP-linked secondary antibody.

### 2.12 Role of ITGB1 in the Regulation of HSF Cell Performance by PvSRA

To verify whether ITGB1 mediates the regulation effect of PvSRA on HSF, before PvSRA treatment, HSF were pretreated with anti-human ITGB1 antibody (1:50) for 1 h at room temperature to block the ITGB1 antigenic site on the membrane surface. Besides, before antibody treatment or test sample treatment, serum starvation was performed. Briefly, after HSF were seeded and adhered to the well, the complete medium was replaced with basal culture medium containing 1% serum and incubated overnight. The concentration of PvSRA was set as 50 μg/ml.

#### 2.12.1 Cell Binding

Binding between PvSRA and HSF was determined by flow cytometry. After the HSF was pretreated with anti-human ITGB1 antibody, the following procedure was the same as abovementioned.

#### 2.12.2 Cell Migration

Transwell migration assay was applied to test the effect of PvSRA on HSF migration. A total of 1 × 10^4^ cells in 100 μl of serum-free medium were added onto the top of the transwell, and 0.6 ml of medium containing the test sample was added into the lower chamber. Migration was allowed to proceed for 24 h in the incubator. Non-migrating cells were removed from the top surface of each membrane with a cotton swab, and then, the membranes were fixed in methanol and stained with 0.1% crystal violet for 30 min at room temperature. After the membranes were washed with PBS, cells that migrated were observed and recorded with inverted microscope. Quantification assay was further applied. Briefly, acetic acid was diluted to 33% (v/v) with ddH_2_O. The bound crystal violet was eluted by adding 400 μl of 33% acetic acid into each insert and shaking for 10 min, the absorbance of the eluent at 590 nm was measured using a plate reader.

#### 2.12.3 Cell Proliferation

CCK8 test was performed to assay the effect of PvSRA on the proliferation of HSF. In brief, HSF cells (6 × 10^3^ cells per well in 100-μl medium) were seeded in 96-well plates and incubated with test samples for 48 h. Then, 10 μl of CCK8 solution was added in each well and incubated for another 2 h. The absorbance was determined at 450 nm by using a Multiskan GO microplate reader. The proliferation ratio was calculated by using the following formula: Proliferation ratio (%) = OD450_test sample_/OD450_blank control_ × 100.

#### 2.12.4 Cell Cycle

HSF were plated in 12-well flat-bottomed tissue culture plates at a density of 3 × 10^5^ cell per well. The cells were treated with PvSRA for 48 h, then collected, and washed with PBS. Thereafter, the cells were fixed with pre-cooled 75% alcohol overnight at 4°C and stained with FxCycle PI/PNase staining solution for 20 min at room temperature. Finally, the cells were analyzed by FACScan, and cell cycle of each group was determined by FlowJo v10.

#### 2.12.5 Cell Cytoskeleton

HSF were plated in 24-well flat-bottomed tissue culture plates at a density of 2 × 10^4^ cells per well. The cells were treated with PvSRA for 48 h, then washed with pre-warmed PBS, and fixed with 3.7% methanol-free formaldehyde solution in PBS for 15 min at room temperature. Further permeabilization was required by using 0.1% Triton X-100 in PBS for 15 min. Thereafter, the cells were blocked with 5% bovine serum albumin for 10 min. Subsequently, FITC phalloidin was added and incubated for 1 h at room temperature. After removing residual staining reagents, cell cytoskeleton was observed and recorded by inverted fluorescence microscope.

#### 2.12.6 Cell Signaling Pathway

Cells were plated in six-well flat-bottomed tissue culture plates at a density of 5 × 10^5^ cells per well. The cells were treated with PvSRA for 24 h. Thereafter, cell lysates were separated through 10% SDS-PAGE, and phosphorylation levels of key enzymes in the MAPK signaling pathways and FAK expression level were analyzed by WB. In this test, anti–phospho-MAPK family, anti-MAPK family, and anti-FAK were used as primary antibodies, whereas anti-GAPDH was used as an internal reference.

#### 2.12.7 Specific Protein Expression

Protein expression of specific DEGs involved in focal adhesion, extracellular matrix, and actin cytoskeleton from RNA-seq results was verified with WB, FACS, or ELISA, and the cutoff of these DEGs was set as log_2_ fold change greater than 1 or less than −1. In this work, anti–MMP-1, anti-CPA3, anti-TFPI2, and anti-MYH3 were used for WB, APC–anti-ITGA8 was used for FACS, and human COMP ELISA Kit was used to quantify COMP concentration in the culture supernatant. In addition, PD-L1 on HSF surface was also tested by FACS.

#### 2.12.8 Statistical Analysis

Data analysis was performed using SPSS 19.0 software (SPSS, Inc., Chicago, IL, United States). All data were presented as mean ± standard error of the mean. Student’s *t*-test was used for inter-group comparison. Statistical significance was considered at *p* < 0.05.

## 3 Results

### 3.1 Expression and Characterization of PvSRA

PvSRA, predicted as an extracellular protein, was identified for its specific antigenic profiling from *P. vivax* erythrocytic stage proteins in our preliminary work, and recombinant PvSRA protein with a molecular weight of 40 kDa was successfully expressed, purified, and verified with anti 6×His-tag antibody, sera from *P. vivax*–infected patients, and specific sera from immunized rabbit or mouse (Figure 1B). Protein array analysis showed that *P. vivax*–infected patients showed significantly higher MFI of total IgG against PvSRA than that from healthy individuals (Figure 1C, *p* < 0.0001). The prevalence for anti-PvSRA antibodies showed a sensitivity of 51% (MFI value of 49 out of 96 patient samples > cutoff value 5,169) and specificity of 96.9% (MFI value of 93 out of 96 healthy samples <5,169), indicating that the recombinant PvSRA protein is specific to serum from *P. vivax*–infected patients. For the positive control PvMSP1-19 (Supplementary Figure 2), the prevalence for anti–PvMSP-19 antibodies showed a sensitivity of 80.2% (MFI value of 77 out of 96 patient samples > cutoff value 5,169) and specificity of 95.8% (MFI value of 92 out of 96 healthy samples < 5,169). Furthermore, PvSRA was detected on the Pv-iRBC membrane in the trophozoite and mature schizont stages of *P. vivax* parasites (Figure 1D).

### 3.2 PvSRA Could Bind With HSF

**FIGURE 2:**
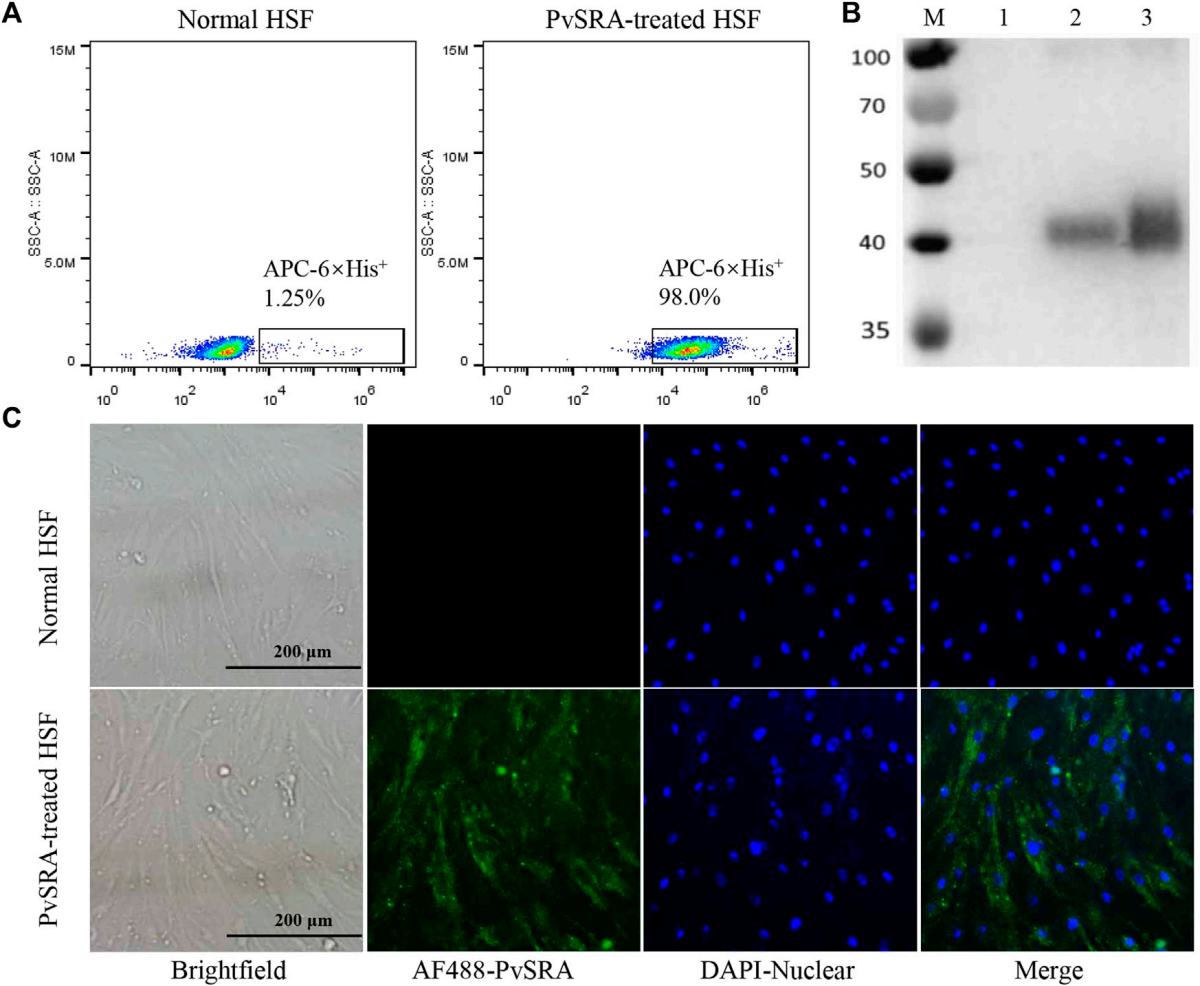
Binding of PvSRA with HSF. The binding of the recombinant PvSRA protein and HSF was detected by using anti-6×His tag antibody in the following tests. **(A)** FACS was applied to test the binding capacity of PvSRA on HSF membrane surface. **(B)** WB analysis of the binding capacity of PvSRA with HSF. M: Protein marker; lane 1: normal HSF lysate; lane 2: PvSRA treated HSF lysate; lane 3: recombinant PvSRA control-sample. **(C)** Immunofluorescence was applied to test the binding between PvSRA and HSF by using anti-6×His tag antibody and Alex Flour 488- conjugated goat anti-rabbit IgG. Nuclei are visualized with DAPI in merged images. Bars represent 200 μm.

Primary results of FACS and WB analyses revealed the presence of 6×His-tag in PvSRA-treated group (Figures 2A,B, respectively), indicating that PvSRA could bind with HSF. In addition, similar observation of the binding between PvSRA and HSF was also noted on HSF membrane surface in PvSRA treatment group through the fluorescence of Alexa Fluor 488–6×His-tag (Figure 2C).

### 3.3 Identification of DEGs in PvSRA-Treated HSF

**FIGURE 3:**
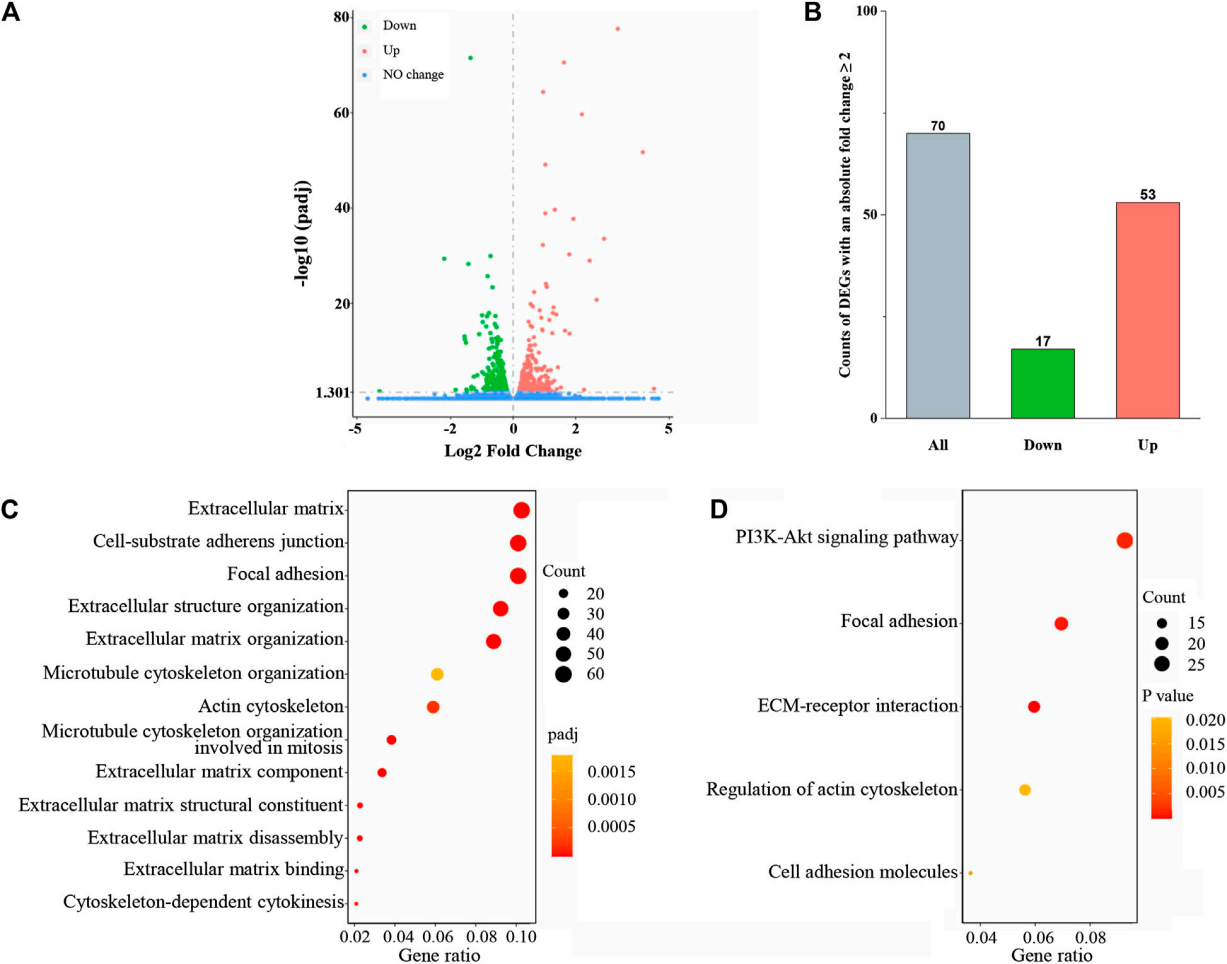
PvSRA significantly induced gene expression changes in HSF. **(A)** Volcano plot of the DEGs. **(B)** Histogram of the count of DEGs with an absolute fold change ≥2. **(C)** GO enrichment analysis of the DEGs obtained from RNA-seq. **(D)** KEGG enrichment analysis of the DEGs obtained from RNA-seq.

RNA-seq was used to detect DEGs between normal HSF and PvSRA-treated HSF, and 24,674 genes were detected (Supplementary Table 1). Among these genes, the expression levels of 639 DEGs changed with a padj less than 0.05, including 337 downregulated DEGs and 302 upregulated DEGs (Figure 3A). Seventy of these DEGs have an absolute fold change ≥2, including 17 downregulated DEGs and 53 upregulated DEGs (Figure 3B). These DEGs are also listed in Supplementary Table 1. The heatmap of DEG expression is supplied as Supplementary Figure 3.

GO and KEGG pathway enrichment analyses were further applied to the abovementioned DEGs to explore the HSF performance changes by PvSRA treatment. As a functional classification system, GO can be divided into three parts: MF, BP, and CC, and the results showed that there were 356 GO terms with padj <0.05, including 56 terms in MF, 224 terms in BP, and 76 terms in CC. Besides, KEGG enrichment analysis results showed that there were 20 pathways with *p* < 0.05 (Supplementary Table 1). A part of these results is displayed in Figures 3C,D. Among these issues, the changes of extracellular matrix and actin cytoskeleton of HSF seemed to agree with the hypothesis that *P. vivax* immune evasion in the spleen is mediated by affecting HSF. Besides, focal adhesion is a type of adhesive contact between the cell and the extracellular matrix through the interaction of the transmembrane protein integrins with their extracellular ligands, and intracellular multiprotein assemblies connect to the actin cytoskeleton. It is well known that focal adhesion plays an essential role during cell BPs, such as cell motility, cell proliferation, and cell survival, because focal adhesions anchor the cell to the substratum and can mediate mechanical and biochemical signaling (Mitra et al., 2005; Burkin et al., 2013). Hence, in this study, we focused on these three issues.

### 3.4 PPIs Analysis of PvSRA

**FIGURE 4:**
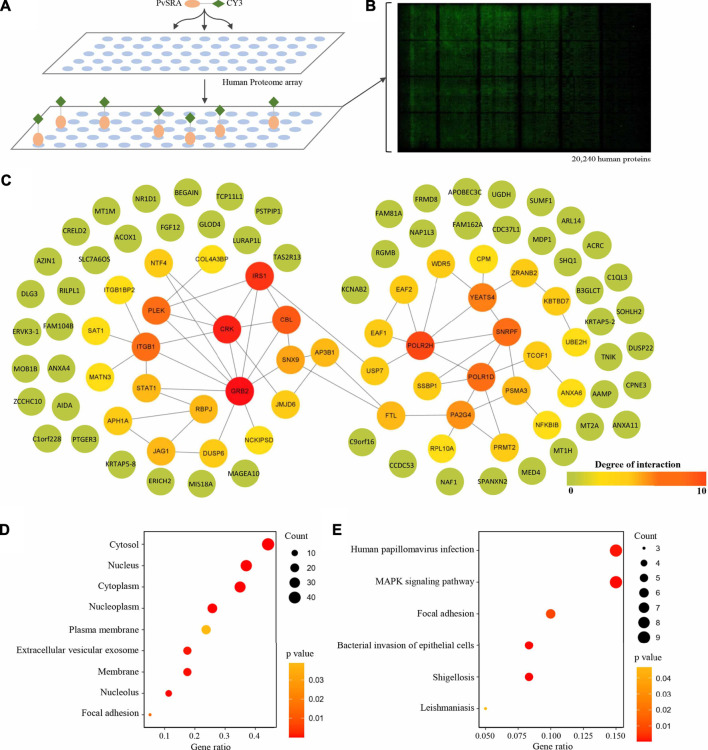
Identification of PvSRA substrates. **(A)** Overall scheme to identify the PvSRA substrates using HuProt arrays. PvSRA labeled with CY3 was added to the array, whereas BSA labeled with CY3 was taken as negative control. **(B)** A representative image of the HuProt array. The reaction between PvSRA and the immobilized protein emits a fluorescent signal. **(C)** PPI network image of the PvSRA substrates. The network was drawn by using STRING program, and the degree of interaction was calculated by Cytoscape software. **(D)** GO enrichment analysis of the PvSRA substrates. **(E)** KEGG pathway enrichment analysis of the PvSRA substrates.

In this study, protein microarray technology was used to predict the potential function of PvSRA through PPIs analysis (Figure 4A). A total number of 97 positive human substrates of PvSRA were preliminarily detected (Figure 4B, Supplementary Table 2), and their interacting network is shown in Figure 4C.

As shown from the results of GO enrichment analysis (Figure 4D), these substrates are located in most of the cell component, including extracellular vesicular exosome and focal adhesion, and this was consistent with the results obtained from RNA-seq. Among these substrates, 21 were found to be involved in 35 meaningful statistical KEGG pathways (Supplementary Table 2). A part of these results is displayed in Figure 4E. Focal adhesion was still in this list, indicating its important role during PvSRA functional process. Notably, some exogenous infections, including human papillomavirus infection, Epstein–Barr virus infection, shigellosis, leishmaniasis, and bacterial invasion of epithelial cells, were found to be in this list.

### 3.5 ITGB1 Is a Receptor of PvSRA on the HSF Cell Surface

**FIGURE 5:**
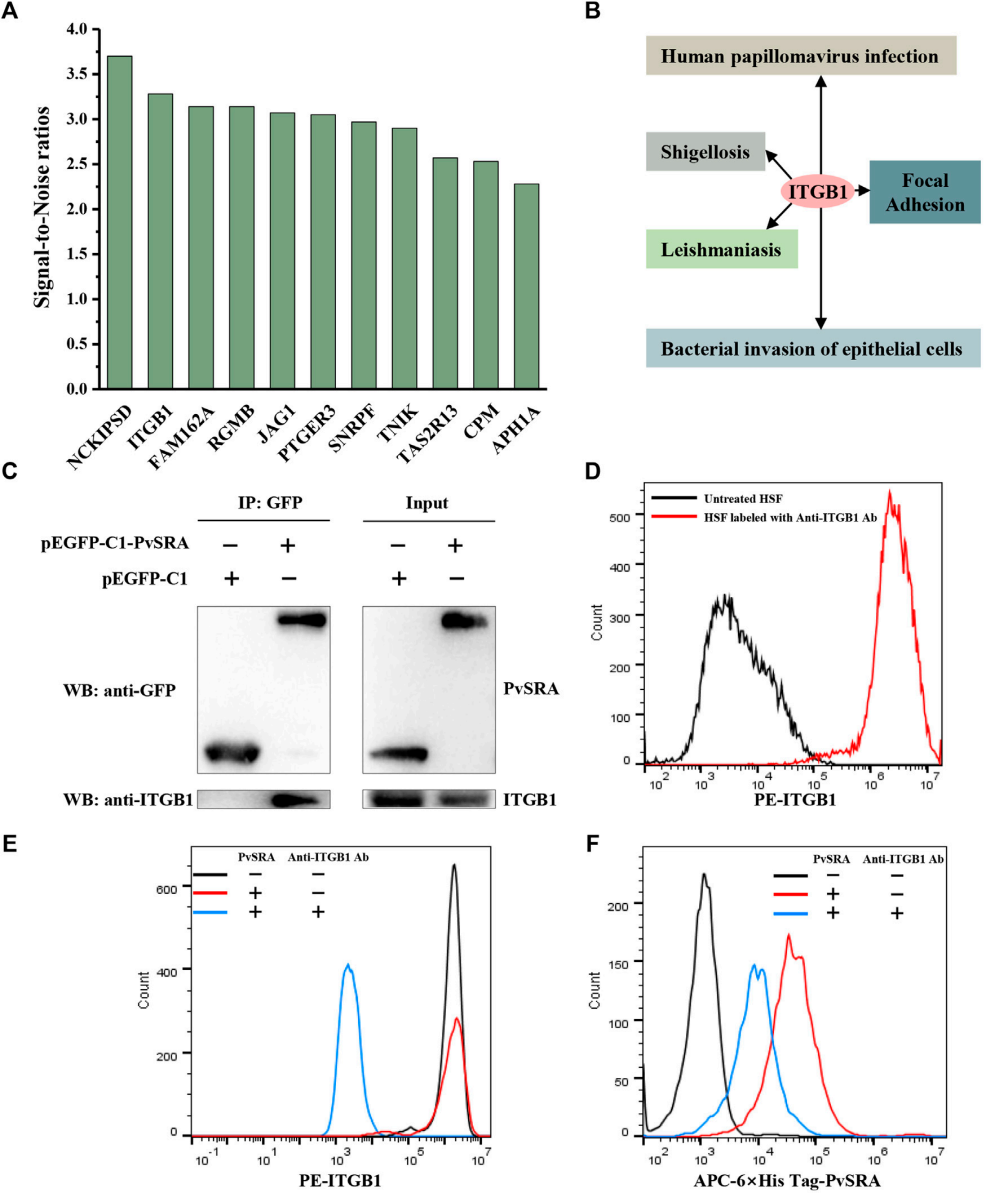
ITGB1 located on HSF cell surface is a receptor for PvSRA. **(A)** Histogram of the PvSRA substrates on cell membrane surface. SNR, the signal-to-noise ratio. **(B)** KEGG analysis demonstrated that ITGB1 is involved in several pathways with a *p* value < 0.05. **(C)** Interaction between PvSRA and ITGB1 was verified by Co-immunoprecipitation testing. ITGB1 is expressed by HEK293 cells, pEGFP- C1- PvRSA was transfected into the HEK293 cells with Lipofectamine 3000, while pEGFP-C1 was used as negative control. **(D)** FACS analysis was performed to verify ITGB1 expression on HSF cell surface. **(E,F)** Backward verification was applied to verify whether ITGB1 is a receptor of PvSRA through blocking ITGB1 function on HSF surface with anti-ITGB1 antibody. Anti-6×His tag antibody and anti-ITGB1 antibody were used to reveal PvSRA and ITGB1 proteins, respectively.

Eleven of the PvSRA substrates are located on the cell membrane (Figure 5A). However, although NCKIPSD is the top of the substrates list according to the SNR, its degree of interaction was much less than that of ITGB1 (Figure 4C), and it was not involved in KEGG enrichment pathways (Supplementary Table 2). More interestingly, ITGB1 is involved in many enriched KEGG pathways, including focal adhesion and several exogenous infections (Figure 5B). The interaction between PvSRA and ITGB1 was further confirmed by co-immunoprecipitation in HEK293 cells (Figure 5C). In addition, FACS results showed that ITGB1 was highly expressed on HSF cell surface (Figure 5D). Moreover, PvSRA could notably bind to the HSF surface, whereas anti-ITGB1 antibody could weaken this binding capability (Figures 5E,F). All these evidenced that ITGB1 on the HSF cell surface is a receptor of PvSRA. Therefore, further investigations were performed to explore the role of ITGB1 during the regulation effect of PvSRA on HSF cell performance.

### 3.6 PvSRA Might Regulate HSF Cell Performance by Interacting With ITGB1

**FIGURE 6:**
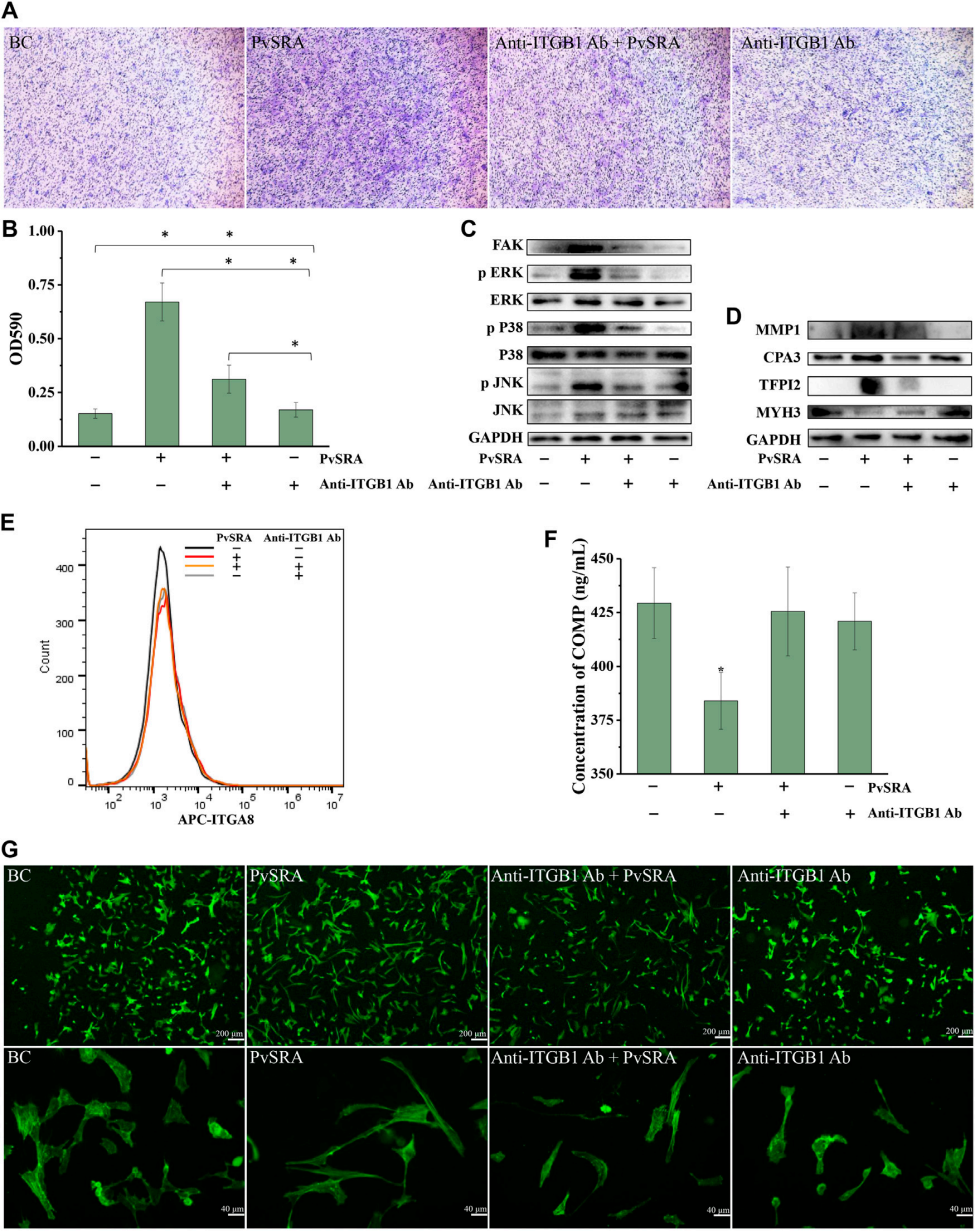
PvSRA attracted migration of HSF and participated in the changing of focal adhesion, extracellular matrix, and actin cytoskeleton of HSF through PvSRA–ITGB1 axis. **(A)** Transwell method was applied to test the effect of PvSRA on HSF migration *in vitro*. **(B)** Quantification assay of the effect of PvSRA on HSF migration by absorptiometry. Asterisk (*) represents that it has statistic difference between groups. **(C)** WB analysis of the expression of FAK and MAPK of PvSRA-treated HSF. **(D)** WB analysis of protein expression of the representative DEGs that were involved in extracellular matrix, focal adhesion, and actin cytoskeleton of PvSRA-treated HSF. **(E)** Expression of ITGA8 on HSF cell surface was tested by FACS. **(F)** COMP secretion was tested by Elisa method. Asterisk (*) represents that it has statistic difference with other groups in same phase. **(G)** The effect of PvSRA on HSF cell cytoskeleton was observed by using FITC phalloidin.

Cell migration is a central process in the development and maintenance of multicellular organisms. In this study, high proportion of HSF migrated to the lower chamber in the PvSRA-treated group when compared with the blank control group (Figure 6A), indicating that PvSRA might attract HSF migration from the top of the transwell to the lower chamber. Besides, the results of quantification assay showed that, when comparing with blank control group, the absorbance of the PvSRA-treated group was increase from 0.152 ± 0.022 to 0.670 ± 0.088, further confirming that PvSRA could attract HSF migration. However, this phenomenon was weakened by pretreating HSF with anti-ITGB1 antibody (Figures 6A,B).

FAK is a kinase that localizes to focal adhesion and regulates signaling in focal adhesion (Schaller, 2010). WB analysis showed that FAK expression of HSF was notably enhanced by PvSRA, whereas this enhancement effect was inhibited by pretreating HSF with anti-ITGB1 antibody (Figure 6C). The results also showed that the protein expression of phospho-MAPK, namely, phospho-p38, phospho-ERK, and phospho-JNK, was increased by PvSRA, whereas the changes in total MAPK expression were slight, indicating that the MAPK pathway was activated by PvSRA treatment. Similarly, this activation effect was also inhibited by anti-ITGB1 antibody pretreatment (Figure 6C).

RNA-seq results showed that PvSRA might affect the focal adhesion, extracellular matrix, and actin cytoskeleton of HSF. Thus, the DEGs involved in these three issues, supplied as Supplementary Figure 4, with log_2_ fold change less than −1 or greater than 1 were used as the potential biomarkers for the changes of HSF by PvSRA treatment in protein expression validation. Among these genes, MMP1, CPA3, and TFPI2 are involved in the extracellular matrix; COMP is involved in the extracellular matrix and focal adhesion; MYH3 is involved in actin cytoskeleton; and ITGA8 is involved in focal adhesion and actin cytoskeleton (Supplementary Figure 4). In relation to the change of their gene expression, the protein expression of MMP1 and TFPI2 was enhanced by PvSRA, and MYH3 expression was downregulated (Figure 6D). However, this effect was partly inhibited by anti-ITGB1 antibody pretreatment. The protein expression of CPA3 was almost not influenced by PvSRA treatment (Figure 6D), and the ITGA8 expression on the HSF surface also showed no change compared with the blank control (Figure 6E). COMP is a secretory protein, and its expression was quantified by ELISA. In comparison with the untreated group, the result showed that the COMP secretion of HSF was significantly decreased from 429.38 ± 16.47 ng/ml to 384.05 ± 20.64 ng/ml after PvSRA treatment (*p* < 0.05), indicating that PvSRA could inhibit COMP secretion. However, this inhibitory effect of PvSRA was weakened by anti-ITGB1 antibody pretreatment (Figure 6F).

FITC phalloidin was used to observe the HSF actin cytoskeleton (Marchesi et al., 2014). As shown from the recorded fluorescence images (Figure 6G), the actin fibers of PvSRA-treated HSF were more stretched, and the cell size was larger compared with those of untreated HSF, indicating that PvSRA treatment could result in HSF cytoskeleton remodeling. However, after HSF were pretreated with anti-ITGB1 antibody, the effect of PvSRA on the HSF cytoskeleton nearly disappeared.

**FIGURE 7:**
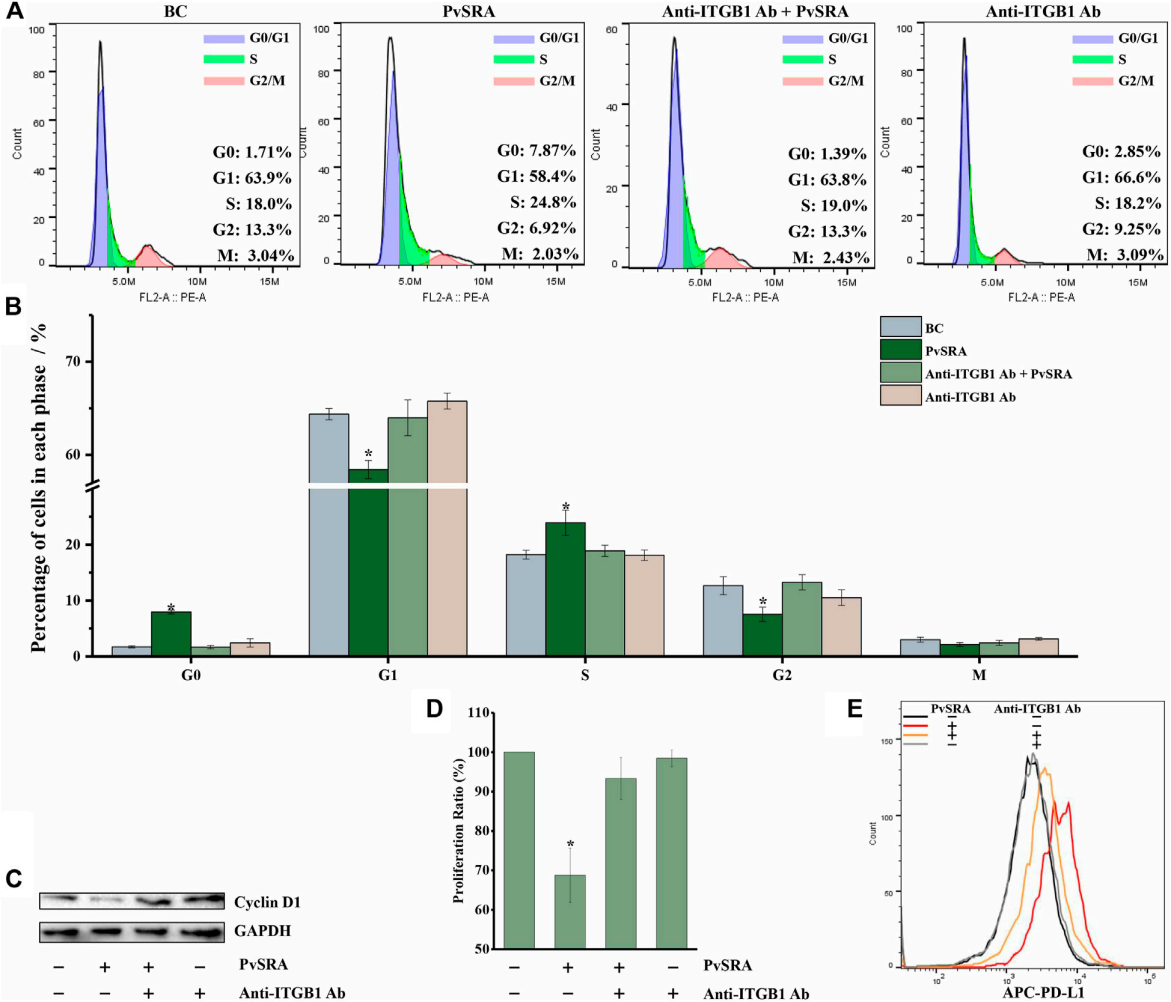
PvSRA has a negative effect on HSF cell cycle and increased PD-L1 expression on HSF surface through PvSRA–ITGB1 axis. **(A)** The effect of PvSRA on HSF cell cycle was tested by FACS. **(B)** Histogram of the changes of cell ration in different cell cycle phase. Asterisk (*) represents that it has statistic difference with other groups in same phase. **(C)** WB analysis was used to test the expression of cell cycle–related protein Cyclin D1. **(D)** CCK8 test was applied to assay the effect of PvSRA on HSF proliferation. Asterisk (*) represents that it has statistic difference with other groups in same phase. **(E)** FACS was applied to test the PD-L1 expression on HSF cell surface.

When compared with untreated HSF, the results of cell cycle analysis showed that the ratio of G0 phase cells in the PvSRA-treated group increased from 0.97 ± 1.23% to 8.94 ± 0.92% (*p* < 0.05), the ratio of S phase cells increased from 17.94 ± 1.24% to 25.42 ± 1.92% (*p* < 0.05), the ratio of G2 phase cells decreased from 12.96 ± 0.89% to 6.76 ± 1.86% (*p* < 0.05), the ratio of G1 phase cells decreased from 64.86 ± 1.37% to 56.46 ± 2.01% (*p* < 0.05), and the ratio of M phase cells was not affected (Figures 7A,B). This phenomenon demonstrated that PvSRA could induce cell cycle arrest. WB analysis showed that the protein expression of Cyclin D1, which plays an essential role in promoting entry into the cell cycle, was downregulated by PvSRA (Figure 7C). Accordingly, CCK test also showed that the cell proliferation ratio decreased to 68.75 ± 6.87% (Figure 7D, *p* < 0.05). However, these effects of PvSRA on HSF cell cycle or proliferation could be weakened by anti-ITGB1 antibody.

In addition, the FACS results showed that PvSRA treatment increased the PD-L1 expression on HSF surface, whereas this effect was also weakened by anti-ITGB1 antibody pretreatment (Figure 7E).

In all the cell experiments, no difference was observed between the blank control group and the group that was only treated with anti-ITGB1 antibody. All these results indicated that PvSRA might regulate HSF cell performance by interacting with ITGB1 that located on the HSF cell surface.

## 4 Discussion

*P. vivax* is one of the most widely distributed human malaria parasites, and the risk of acute *P. vivax* attacks after splenectomy is increased, which even outweighs the risk of *P. falciparum* attack (Kho et al., 2019). However, mechanisms that underline immune evasion in *P. vivax* infection remain unclear. A hypothesis holds that Pv-iRBC can selectively cytoadhere to splenic fibroblasts and cause spleen structural remodeling and resulting in immune evasion from spleen clearance (del Portillo et al., 2004). In the present study, we provide evidence that PvSRA, a newly found exported protein from *P. vivax* to the surface of infected erythrocyte membrane, could interact with HSF and induce cell performances changes of HSF through interaction with ITGB1 on HSF cell surface.

As a blood filter, the spleen is mainly composed of the red pulp, white pulp, and their boundary called the marginal zone. Under normal circumstances, abnormal or aged erythrocytes are typically removed by the spleen through phagocytosis or antigen-specific immune response. However, it was found that large numbers of Pv-iRBC in the red pulp (Machado Siqueira et al., 2012), indicating that *P. vivax* could pass through endothelial slits, enter the spleen, and avoid the immune clearance of spleen (Handayani et al., 2009; Machado Siqueira et al., 2012). The rodent malaria *P. yoelii* has been extensively used to study molecular aspects of virulence in malaria, such as pre-erythrocytic stages of infection and pathogenesis of erythrocytic stages, mainly because of the existence of strains with different cellular tropisms, growth curves, and clinical outcomes, such as the reticulocyte-prone non-lethal *P. yoelii* 17X strain and the normocyte-prone lethal *P. yoelii* 17XL strain. Among these two strains, *P. yoelii* strain 17X infections in mouse spleen are similar to *P. vivax* infections in human spleen. Researchers used the Balb/c *P. yoelii* rodent malaria model to study the function and structure of the spleen, and they noticed lower numbers of parasites within macrophages in infections with 17X than in infections with 17XL. However, because these numbers were observed together with larger numbers of parasites in spleens infected with 17X and no differences were observed in either *in vitro* or *in vivo* phagocytic activity between the two infections, it can be assumed that the differences in the numbers of parasites within the macrophages are not due to an increased ability of macrophages to kill parasites during non-lethal infections. This finding provides further evidence of different spleen compartmentalization in animals infected with non-lethal strains (Martin-Jaular et al., 2011). Besides, it was found that non-lethal *P. yoelii* 17X strain induces remodeling of the spleen through the formation of a spleen tissue barrier of fibroblastic origin, causing the “open” circulation of the spleen to suddenly and temporarily change into a “closed” circulation (Weiss, 1989). Such barrier facilitates the channeling of blood from arterioles to venules and the adherence of infected reticulocytes to it thus physically protecting them from destruction by macrophages (Martin-Jaular et al., 2011). Because of similarities between *P. yoelii* strain 17X infections in BALB/c mice and *P. vivax* infections, a hypothesis holds that Pv-iRBC induces a spleen blood barrier of fibroblastic origin to which infected reticulocytes adhere in facilitating macrophage clearance escape (del Portillo et al., 2004).

HSF, which is the fibroblastic origin mentioned above, also known as splenic fibroblastic reticular cells, significantly contribute to the immunity of the organism because they augment the reticular cell fiber filtration beds of spleens when stressed by infection, imperfect erythrocytes, or immunological deficiency. Thus, they play essential roles in splenic clearance of the blood (Weiss, 1991). In addition, HSF provide a three-dimensional (3D) microenvironment and set up a niche for lymphoid cells, including T, B, and dendritic cells, through the expression of chemoattractants. This niche not only supports the onset and perpetuation of immune responses but also activates, enhances, and even inhibits immune responses (Mueller et al., 2007; Lukacs-Kornek et al., 2011). A recent study showed that plasma-derived extracellular vesicles from *P. vivax* patients are preferentially uptaken by HSF as compared with the uptake of extracellular vesicles from healthy individuals. After this uptake, Pv-iRBC has shown specific adhesion properties to these HSF (Toda et al., 2020). For extracellular vesicles that act as shuttle vectors or signal transducers that can deliver specific biological information and have progressively emerged as key regulators of organized communities of cells within multicellular organisms in health and disease (Simeone et al., 2020), we suspected that the cross-talk between Pv-iRBC and HSF might contribute to the immune evasion of *P. vivax*.

It is well known that variant surface antigens of *P. falciparum*, such as PfEMP1, play essential role during its immune evasion process. For instance, it mediates binding of infected erythrocytes to uninfected erythrocytes, and it is thought that *P. falciparum* evades the host immune system by shielding the infected erythrocytes and the newly released merozoites from host invasion-inhibitory antibodies in this way (Yam et al., 2017). Besides, PfEMP1 have also been found to interact with a variety of cell surface receptors on the endothelial cells, resulting in the adhesion of Pf-iRBC to endothelium (Yam et al., 2017). In addition, PfEMP1 is anchored at the erythrocytes membrane skeleton by knobs, macromolecular complexes that consist of knob-associated histidine-rich protein (KAHRP), whereas KAHRP binding with the erythrocyte membrane skeleton leads to an increased rigidity and further decreased the deformability of Pf-iRBC (Maier et al., 2008). Therefore, unlike normal erythrocytes that could pass through blood vessel wall into the spleen circulation through their deformability, Pf-iRBCs that adhered to endothelium are unable to enter the spleen, allowing *P. falciparum* to avoid immune clearance by the spleen (Chen et al., 2000; Turner et al., 2013). These findings showed that the exported antigen from plasmodium on infected erythrocyte membrane surface might play important role during immune evasion.

PvSRA, predicted as an extracellular protein, is a newly found erythrocytic stage antigen identified in our study, and it was confirmed as a merozoite surface protein that could be exported to the surface of infected erythrocyte membrane. Besides, *in vitro* cell experiment revealed that PvSRA could bind with HSF, indicating that PvSRA might mediate the binding of Pv-iRBC to the splenic fibroblasts in spleen. Considering the abovementioned *P. vivax* immune evasion hypothesis and the important role of exported antigen from plasmodium in the erythrocytic stage (Chen et al., 2000), we suspected that PvSRA might be involved in *P. vivax* immune evasion in the spleen. Because RNA-seq is a high-throughput technology used to provide a comprehensive view of entire transcriptome, and it enables a wide range of applications such as the discovery of novel genes, gene/transcript quantification, and differential expression and functional analysis (Wang et al., 2009; Wang et al., 2019), this technology was applied to test our speculation. The results showed that the expression of 639 of 24,647 genes was significantly regulated by PvSRA. GO and KEGG pathway enrichment analyses manifested that these DEGs are involved in various terms and signaling pathways. Interestingly, focal adhesion, extracellular matrix, and actin cytoskeleton related terms or pathway were founded in the enrichment results. Among them, focal adhesion, a specialized structure formed at the cell–extracellular matrix contacts points, where bundles of actin filaments are anchored to transmembrane receptors of the integrin family through a multi-molecular complex of junctional plaque proteins. Focal adhesion structurally links the cytoskeleton to the extracellular matrix and transmits mechanical signals (Mitra et al., 2005; Burkin et al., 2013). Thus, it not only plays an essential role during cell motility through the regulation of the actin cytoskeleton but also is involved in the regulation of other cell functions, such as cell proliferation and survival, through signal transmission (Mitra et al., 2005; Burkin et al., 2013). Extracellular matrix is a non-cellular 3D macromolecular network composed of collagens, proteoglycans/glycosaminoglycans, elastin, fibronectin, laminins, and several other glycoproteins. These components bind each other as and cell adhesion receptors forming a complex network into which cells reside in all tissues and organs. Cell surface receptors transduce signals into cells from extracellular matrix, which regulate diverse cellular functions, such as survival, growth, migration, and differentiation, and are vital for maintaining normal homeostasis, whereas the progression of several pathologic conditions is associated with the negative deregulation of extracellular matrix composition and structure (Theocharis et al., 2015). The actin cytoskeleton, a collection of actin filaments with their accessory and regulatory proteins, is the primary force-generating machinery in the cell. It was found that its generated force plays essential role in cell migration, cell shape, and mechanical properties of the cell surface, drive the intracellular motility and morphogenesis of membrane organelles, and allow cells to form adhesions with each other and with the extracellular matrix (Svitkina, 2018). Therefore, the changes of these three aspects of HSF seemed to agree with the hypothesis that *P. vivax* immune evasion in the spleen is mediated by affecting HSF.

It is well known that proteins rarely act alone as their functions tend to be regulated. Many molecular processes are carried out by molecular machines that are built from many protein components organized by their PPIs; however, abnormal or aberrant PPIs could cause diseases (Ryan and Matthews, 2005). Therefore, analyzing PPIs can help identify novel pathways to gain basic knowledge of the physiological activity or pathological action. Moreover, it can also help find new therapeutic targets of diseases and predict the potential function of newly found or unknown proteins (Berger and Iyengar, 2009). Protein microarray is a high-throughput method used not only to track interactions and activities of proteins but also to determine proteins function. Therefore, this technology was applied in the present work to search for substrates of PvSRA and explore the potential function of PvSRA through PPIs analysis. The results showed that substrates of PvSRA were not only distributed in different regions of the cell, such as membrane, cytoplasm, and nucleus, but also located at focal adhesion sites and extracellular vesicular exosome, indirectly indicating that PvSRA is capable of binding with or entering into some host cells through PPIs. To determine whether PvSRA induced HSF gene expression changes through protein interaction with some unknown substrates or receptors on the HSF membrane surface, we mainly focused on the PvSRA substrates located on the cell membrane surface in the present work. Among the surface receptors of PvSRA, ITGB1 was observed for its unique properties during BPs. Integrin family members are membrane receptors involved in cell adhesion and recognition in various processes including embryogenesis, hemostasis, tissue repair, and immune response (Wu et al., 2010). They link the actin cytoskeleton with the extracellular matrix and transmit signals bidirectionally between the extracellular matrix and cytoplasmic domains (Nix and Beckerle, 1997). One of the members of the integrin family, ITGB1, has several functions, including actin binding, cadherin binding, cell adhesion molecule binding, collagen binding involved in cell-matrix adhesion, fibronectin binding, laminin binding, protease binding, and protein tyrosine kinase binding (Mueller et al., 1999; Moog-Lutz et al., 2003; Cerecedo et al., 2006; Guo et al., 2014; Sipila et al., 2014; Zhao et al., 2016). Hence, it appears to be an important receptor of cellular adhesion molecules because it mediates adhesion between cell and cell or cell and extracellular matrix. In addition, it is primarily responsible for targeting integrin dimers to the appropriate subcellular locations. Function analysis and literature review showed that ITGB1 also participates in extracellular matrix organization, protein localization, cell cycle, cell proliferation and apoptosis, cell migration, and other BPs (Mueller et al., 1999; Miles et al., 2008; Juric et al., 2009; Zhao et al., 2016). In the current study, we confirmed that ITGB1 was expressed on HSF cell surface, indicating that Pv-iRBCs can bind with HSF through PvSRA–ITGB1 interaction and might further influenced HSF cell performance.

ITGB1-regulated subcellular locations in adhesive cells mainly include focal adhesions. In addition, the results of enrichment analysis showed that ITGB1 was involved in several microbial infections as a receptor for viruses, bacteria, and parasites. During these infections, ITGB1 mainly acts as a receptor of microbial composition or a constituent of focal adhesion structure to participate in the invasion process (Figueira et al., 2015; Olafsson et al., 2019). Interestingly, the RNA-seq results showed that the expression of genes involved in the extracellular matrix, focal adhesion, and actin cytoskeleton was significantly changed in the PvSRA-treated HSF group. This could demonstrate that Pv-iRBCs have the potential to induce the formation of focal adhesion structure on the HSF surface through PvSRA–ITGB1 interaction, followed by actin cytoskeleton remodeling and the alteration of HSF function, finally resulting in the escape from spleen clearance*.*

On the above basis of the bioinformation from RNA-seq and PPIs analyses, *in vitro* cell-based validations were further performed to verify the above speculation. The results demonstrated that PvSRA could bind to the HSF surface and attract HSF migration, indicating that Pv-iRBC could adhere to HSF after they enter the spleen. As a kinase that localizes to focal adhesion and regulates signaling in focal adhesion, FAK communicates signals between integrins and intracellular proteins and regulates diverse cellular processes such as cell proliferation, migration, actin cytoskeleton remodeling, and invasion (Rosen and Dube, 2006; Chen J.-S. et al., 2010; Schaller, 2010; Roa-Espitia et al., 2016). We observed that the expression of FAK was significantly enhanced by PvSRA treatment and, the MAPK signal pathway, a downstream signaling event of focal adhesion (Mitra et al., 2005), was also activated, indicating that PvSRA could induce focal adhesion formation on the HSF surface, followed by downstream signal activating. Besides, the results showed that PvSRA treatment induced HSF actin cytoskeleton remodeling, indicating that Pv-iRBC has the potential to alter the original cellular structure of HSF. We further tested protein expression of the representative DEGs involved in focal adhesion, extracellular matrix, and actin cytoskeleton of PvSRA-treated HSF. Among these genes, MMP1 is a prototypical MMP, primarily functioning to degrade collagen types 1 and 3 (Pardo and Selman, 2006). CPA3 is a member of the family of zinc metalloproteases, which are involved in the degradation of endogenous proteins (Roy et al., 2014). TFPI2 may play a role in the regulation of plasmin-mediated matrix remodeling (Du et al., 2003). COMP, an extracellular secreted glycoprotein, may play a role in the structural integrity via its interaction with other extracellular matrix proteins such as collagens and fibronectin (Haudenschild et al., 2011). ITGA8 is a cell surface protein that belongs to the alpha integrin family of transmembrane cell surface receptors, and it requires the formation of a heterodimer with ITGB1 for receptor function (Hung et al., 2018). MYH3 belongs to a group of proteins called myosin, which are involved in cell movement and transport of materials within and between cells (Li et al., 2016). The results from cell-based assays showed that, after PvSRA treatment, the expression of MMP1 and TFPI2 was obviously upregulated, whereas that of MYH3 and COMP was decreased and was consistent with the results obtained from RNA-seq. However, no changes were observed in the expression of CPA3 and ITGA8, indicating that the expression levels of these two genes were inconsistent with their protein expression levels. We also found that PvSRA has a significantly negative effect on the HSF cycle and protein expression of cell cycle–related gene Cyclin D1, which might further result in proliferation inhibition. These negative effect on HSF proliferation indicated that Pv-iRBC might inhibit the proliferation of the barrier cells after they enter the spleen. Besides, for HSF that mainly play its immune role through increasing in number to form splenic filtration beds when spleen is attacked by pathogens (Weiss, 1991), the present finding also indicated that Pv-iRBC might decrease the capacities of the spleen through inhibiting HSF proliferation. In present work, we also found that PvSRA treatment increased PD-L1 expression on the HSF surface. PD-L1 is a protein that allows some cells to escape the attack by the immune system. PD-L1 interacts with PD-1 on some important immune system cells such as T cells. It is acknowledged that PD-1/PD-L1 axis plays a crucial role in the progression of tumor by altering status of immune surveillance (Yi et al., 2018). Except for tumor progression, PD-L1 expressed on infected host cell also plays an essential role during extraneous pathogens invasion or infection process. For example, research has shown that lymphocytic choriomeningitis virus infection targeting of mouse splenic fibroblasts contributes to its chronic infection, because the upregulation expression of PD-L1 on infected splenic fibroblasts prevents severe immunopathology and architectural disruption of splenic fibroblast network in the spleen (Mueller et al., 2007). Because Pv-iRBC also has the potential to target HSF, we suspected that Pv-iRBC might also evade host immune attack in a similar way. These findings revealed that Pv-iRBC has the potential to decrease the immunological capacities of spleen through targeting HSF. However, the results of these *in vitro* experiments showed that the effect of PvSRA on HSF could be inhibited or weakened by blocking ITGB1 receptor on the HSF surface, demonstrating that ITGB1 is indispensable during PvSRA–HSF interaction. On the basis of above findings and in consideration of ITGB1 that was found to be involved in several microbial infections as a receptor for viruses, bacteria, and parasites (Figueira et al., 2015; Olafsson et al., 2019), we suspected that ITGB1 might also be invloved in *P. vivax* immune evasion in the spleen.

**FIGURE 8:**
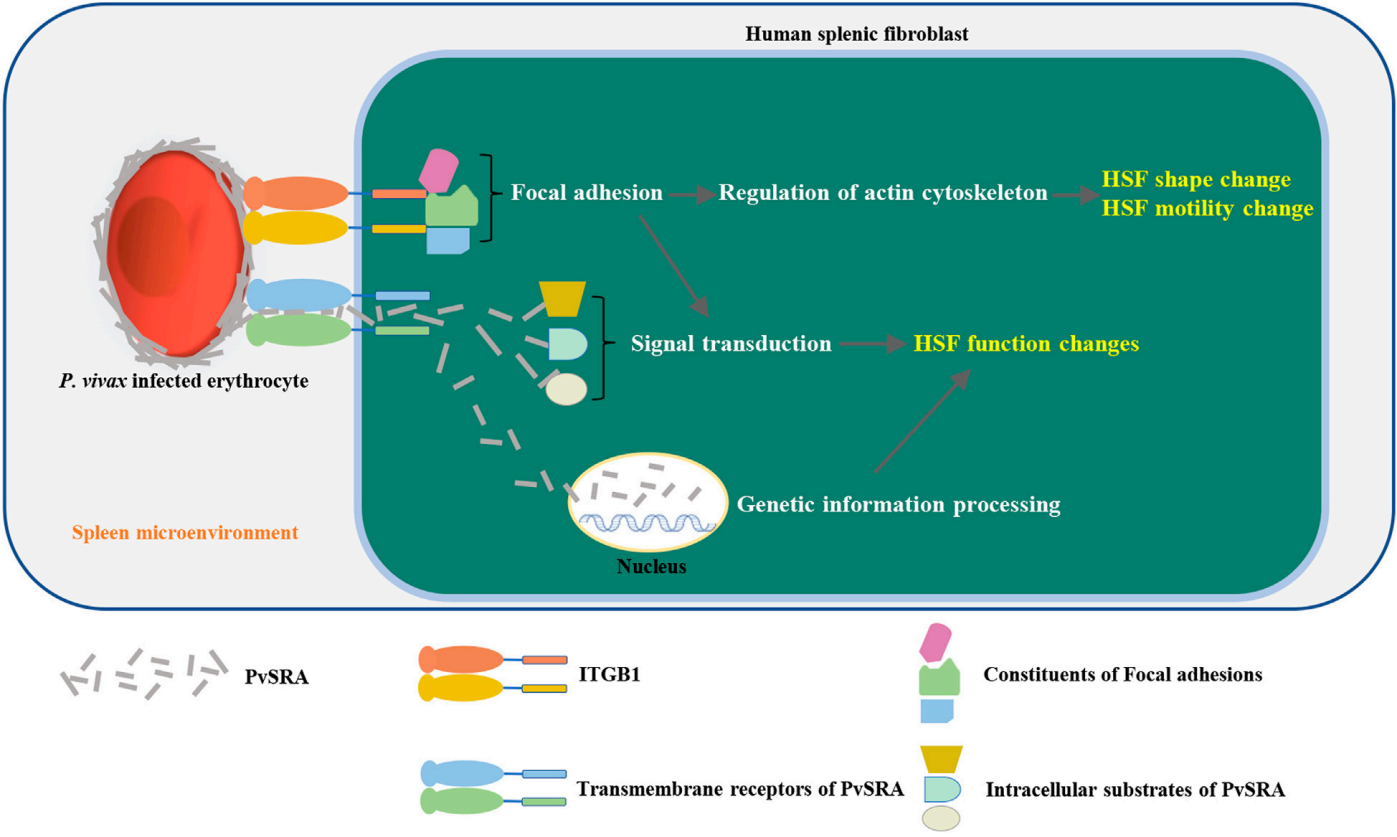
Schematic diagram of the immune evasion hypothesis of *P. vivax* in the spleen. After entering the spleen, *P. vivax*–infected erythrocytes adhere to HSF through ligand (PvSRA)–receptor (ITGB1) axis. This is followed by the formation of the focal adhesion structure on HSF surface, leading to the actin cytoskeleton remodel, and then, the original shape and motility of HSF was affected and changed to induce the formation of barrier cells. Besides, focal adhesion might also modulate the capabilities of HSF by further activating FAK pathway and downstream pathway (i.e., MAPK pathway). In addition, PvSRA might translocated into intracellular environment through other substrates to further affect or interfere the signal transduction or genetic information processing of HSF through other PPIs. This finally would alter the immunoregulation role of HSF in the spleen to result in the immune evasion of *P. vivax*.

In addition to the bioinformation from RNA-seq and PPIs analyses, the results of *in vitro* assays contributed in improving the prediction of immune evasion process of *P. vivax*, which could be summarized as follows: after entering the spleen, Pv-iRBCs adhere to HSF through the ligand (PvSRA)–receptor (ITGB1) axis, followed by the formation of the focal adhesion structure on the HSF surface. Given that some of the constituents of focal adhesion participate in the structural link between membrane receptors and the actin cytoskeleton (Mitra et al., 2005; Burkin et al., 2013), the original shape and motility of HSF were affected and changed, which might induce the formation of barrier cells. Moreover, because some constituents of focal adhesion are signaling molecules (i.e., different protein kinases and phosphatases, substrates, and various adapter proteins) (Mitra et al., 2005; Burkin et al., 2013), focal adhesion might further modulate other capabilities of HSF, such as cell proliferation and extracellular matrix, then activating its downstream pathway (i.e., MAPK pathway). In addition, PvSRA might also have the possibility to enter into the intracellular or endonuclear environment through interacting with other receptors and then further affects or interferes with the signal transduction or genetic information processing of HSF through other PPIs. This would finally alter the immunoregulation role of HSF in the spleen to result in the immune evasion of *P. vivax* (Figure 8).

However, because the present study is mainly based on previous hypotheses and RNA-seq prediction and the prediction was only validated roughly by *in vitro* assays, there are some research limitations in present work. Because *P. vivax* parasites cannot be maintained reliably in continuous cultures *in vitro*, biology research on *P. vivax* faces different challenges. Therefore, some findings or predictions in present work mainly obtained through *in vitro* protein molecular cell experiments, lacking the verification through intercellular interaction experiments between Pv-iRBC and HSF. The recent development allows to purify *P. vivax* parasites in the ring stage and to develop them into late stages (Roobsoong et al., 2015; Prajapati et al., 2019). This will help address our future questions related to surface expression or discharge of PvSRA and to demonstrate a direct binding between Pv-iRBCs and HSF. Besides, because acetone could penetrate cell membranes, PvSRA location on Pv-iRBC membrane surface needed further verification by co-staining with erythrocyte surface proteins (e.g., Band 3 or Glycophorin A) without permeabilizing cells (Auffray et al., 2001). In addition, present research on *P. vivax* immune evasion mechanism is not deep enough; both the high-throughput analysis and *in vitro* validation were superficial. For instance, the present work mainly focused on focal adhesion, extracellular matrix, and actin cytoskeleton changes of HSF, and only partial related genes and pathway were verified *in vitro*; detailed relationship between signal pathway and cell performance changes has not been yet evaluated; the possibility that PvSRA might enter into HSF also needed further verification, and the prerequisite of this speculation is to make sure whether PvSRA could discharge or secrete from Pv-iRBC through testing the presence of PvSRA in the culture medium with ELISA or some other methods when culturing the *P. vivax* parasites *in vitro*. Furthermore, because there have been few related studies about relevant research, no appropriate control samples were included in present work. Previous studies have found that the cytoadhesion of Pv-iRBCs was, in part, mediated by VIR proteins localized at the surface of Pv-iRBCs (Fernandez-Becerra et al., 2009; Carvalho et al., 2010), and a recent research reveals that VIR14 protein (PlasmoDB ID: PVX_108770) mediates specific adhesion to HSF expressing ICAM-1 (Fernandez-Becerra et al., 2020), indicating that these VIR proteins could be taken as control samples in the following studies. Overall, the present study was mainly a predictive work, and the findings may provide a basis for further research in this field.

## Data Availability

The datasets presented in the study are deposited in the Sequence Read Archive (SRA) repository, the accession number is PRJNA776344.

## Supplementary Material

Supplementary Table 1 Quantity analysis of the genetic changes in gene expression between normal HSF and PvSRA-treated HSF by RNA-seq.

Supplementary Table 2 Detecting of the protein substrates of PvSRA by Human proteome microarray analysis.

Supplementary Figure 1 Determination of the use concentration of PvSRA by protein arrays. **(A)** Protein arrays probed with anti–6×His-tag antibody. **(B)** Correlation between spot intensities and the serial diluted purified His-tagged PvSRA protein.

Supplementary Figure 2 Comparison of IgG antibody responses to recombinant proteins PvMSP1-19.

Supplementary Figure 3 The heatmap of genetic changes in gene expression between normal HSF and PvSRA-treated HSF.

Supplementary Figure 4 Expression change of the differentially expressed genes (DEGs) that were involved in **(A)** extracellular matrix, **(B)** focal adhesion, and **(C)** actin cytoskeleton.

## References

[B2] Auffray I. Marfatia S. de Jong K. Lee G. Huang C.-H. Paszty C. (2001). Glycophorin A Dimerization and Band 3 Interaction during Erythroid Membrane Biogenesis: *In Vivo* Studies in Human Glycophorin A Transgenic Mice. Blood 97 (9), 2872–2878. 10.1182/blood.v97.9.2872 11313283

[B3] Bassat Q. Alonso P. L. (2011). Defying Malaria: Fathoming Severe Plasmodium Vivax Disease. Nat. Med. 17 (1), 48–49. 10.1038/nm0111-48 21217683

[B4] Berger S. I. Iyengar R. (2009). Network Analyses in Systems Pharmacology. Bioinformatics 25 (19), 2466–2472. 10.1093/bioinformatics/btp465 19648136 PMC2752618

[B5] Bozdech Z. Mok S. Hu G. Imwong M. Jaidee A. Russell B. (2008). The Transcriptome of Plasmodium Vivax Reveals Divergence and Diversity of Transcriptional Regulation in Malaria Parasites. Proc. Natl. Acad. Sci. 105 (42), 16290–16295. 10.1073/pnas.0807404105 18852452 PMC2571024

[B6] Burkin H. R. Rice M. Sarathy A. Thompson S. Singer C. A. Buxton I. L. O. (2013). Integrin Upregulation and Localization to Focal Adhesion Sites in Pregnant Human Myometrium. Reprod. Sci. 20 (7), 804–812. 10.1177/1933719112466303 23298868 PMC3678871

[B7] Carvalho B. O. Lopes S. C. P. Nogueira P. A. Orlandi P. P. Bargieri D. Y. Blanco Y. C. (2010). On the Cytoadhesion ofPlasmodium Vivax-Infected Erythrocytes. J. Infect. Dis. 202 (4), 638–647. 10.1086/654815 20617923

[B8] Cerecedo D. Mondragon R. Cisneros B. Martinez-Perez F. Martinez-Rojas D. Rendon A. (2006). Role of Dystrophins and Utrophins in Platelet Adhesion Process. Br. J. Haematol. 134 (1), 83–91. 10.1111/j.1365-2141.2006.06120.x 16803572

[B9] Chen J.-H. Jung J.-W. Wang Y. Ha K.-S. Lu F. Lim C. S. (2010a). Immunoproteomics Profiling of Blood Stage Plasmodium Vivax Infection by High-Throughput Screening Assays. J. Proteome Res. 9 (12), 6479–6489. 10.1021/pr100705g 20949973

[B10] Chen J.-S. Huang X.-H. Wang Q. Chen X.-L. Fu X.-H. Tan H.-X. (2010b). FAK Is Involved in Invasion and Metastasis of Hepatocellular Carcinoma. Clin. Exp. Metastasis 27 (2), 71–82. 10.1007/s10585-010-9306-3 20180147

[B11] Chen Q. Heddini A. Barragan A. Fernandez V. Pearce S. F. A. Wahlgren M. (2000). The Semiconserved Head Structure of Plasmodium Falciparum Erythrocyte Membrane Protein 1 Mediates Binding to Multiple Independent Host Receptors. J. Exp. Med. 192 (1), 1–10. 10.1084/jem.192.1.1 10880521 PMC1887712

[B12] Commons R. J. Simpson J. A. Thriemer K. Hossain M. S. Douglas N. M. Humphreys G. S. (2019). Risk of Plasmodium Vivax Parasitaemia after Plasmodium Falciparum Infection: a Systematic Review and Meta-Analysis. Lancet Infect. Dis. 19 (1), 91–101. 10.1016/S1473-3099(18)30596-6 30587297 PMC6300482

[B13] del Portillo H. A. Lanzer M. Rodriguez-Malaga S. Zavala F. Fernandez-Becerra C. (2004). Variant Genes and the Spleen in Plasmodium Vivax Malaria. Int. J. Parasitol. 34 (13-14), 1547–1554. 10.1016/j.ijpara.2004.10.012 15582531

[B14] Douglas N. M. Lampah D. A. Kenangalem E. Simpson J. A. Poespoprodjo J. R. Sugiarto P. (2013). Major burden of Severe Anemia from Non-falciparum Malaria Species in Southern Papua: a Hospital-Based Surveillance Study. Plos Med. 10 (12), e1001575. 10.1371/journal.pmed.1001575 24358031 PMC3866090

[B15] Du X. Deng F.-M. Chand H. S. Kisiel W. (2003). Molecular Cloning, Expression, and Characterization of Bovine Tissue Factor Pathway Inhibitor-2. Arch. Biochem. Biophys. 417 (1), 96–104. 10.1016/s0003-9861(03)00332-1 12921785

[B16] Elizalde-Torrent A. Val F. Azevedo I. C. C. Monteiro W. M. Ferreira L. C. L. Fernández-Becerra C. (2018). Sudden Spleen Rupture in a Plasmodium Vivax-Infected Patient Undergoing Malaria Treatment. Malar. J. 17 (1), 79. 10.1186/s12936-018-2228-2 29433507 PMC5809972

[B17] Fernandez-Becerra C. Bernabeu M. Castellanos A. Correa B. R. Obadia T. Ramirez M. (2020). Plasmodium Vivaxspleen-dependent Genes Encode Antigens Associated with Cytoadhesion and Clinical protection. Proc. Natl. Acad. Sci. USA 117 (23), 13056–13065. 10.1073/pnas.1920596117 32439708 PMC7293605

[B18] Fernandez-Becerra C. Yamamoto M. M. Vêncio R. Z. N. Lacerda M. Rosanas-Urgell A. del Portillo H. A. (2009). Plasmodium Vivax and the Importance of the Subtelomeric Multigene Vir Superfamily. Trends Parasitol. 25 (1), 44–51. 10.1016/j.pt.2008.09.012 19036639

[B19] Figueira C. P. Carvalhal D. G. F. Almeida R. A. Hermida M. d. E.-R. Touchard D. Robert P. (2015). Leishmania Infection Modulates Beta-1 Integrin Activation and Alters the Kinetics of Monocyte Spreading over Fibronectin. Sci. Rep. 5, 12862. 10.1038/srep12862 26249106 PMC4528201

[B20] Guo Z. Neilson L. J. Zhong H. Murray P. S. Zanivan S. Zaidel-Bar R. (2014). E-cadherin Interactome Complexity and Robustness Resolved by Quantitative Proteomics. Sci. Signal. 7 (354), rs7. 10.1126/scisignal.2005473 25468996 PMC4972397

[B21] Handayani S. Chiu D. T. Tjitra E. Kuo J. S. Lampah D. Kenangalem E. (2009). High Deformability ofPlasmodium Vivax-Infected Red Blood Cells under Microfluidic Conditions. J. Infect. Dis. 199 (3), 445–450. 10.1086/596048 19090777 PMC4337984

[B22] Haudenschild D. R. Hong E. Yik J. H. N. Chromy B. Mörgelin M. Snow K. D. (2011). Enhanced Activity of Transforming Growth Factor β1 (TGF-Β1) Bound to Cartilage Oligomeric Matrix Protein. J. Biol. Chem. 286 (50), 43250–43258. 10.1074/jbc.M111.234716 21940632 PMC3234822

[B23] Hung C. F. Wilson C. L. Chow Y.-H. Schnapp L. M. (2018). Role of Integrin Alpha8 in Murine Model of Lung Fibrosis. PLoS One 13 (5), e0197937. 10.1371/journal.pone.0197937 29813125 PMC5973593

[B24] Im J. H. Kwon H. Y. Baek J. Park S. W. Durey A. Lee K. H. (2017). Severe Plasmodium Vivax Infection in Korea. Malar. J. 16 (1), 51. 10.1186/s12936-017-1684-4 28129766 PMC5273855

[B25] Juric V. Chen C.-C. Lau L. F. (2009). Fas-mediated Apoptosis Is Regulated by the Extracellular Matrix Protein CCN1 (CYR61) *In Vitro* and *In Vivo* . Mol. Cell Biol 29 (12), 3266–3279. 10.1128/MCB.00064-09 19364818 PMC2698731

[B26] Kho S. Andries B. Poespoprodjo J. R. Commons R. J. Shanti P. A. I. Kenangalem E. (2019). High Risk of Plasmodium Vivax Malaria Following Splenectomy in Papua, Indonesia. Clin. Infect. Dis. 68 (1), 51–60. 10.1093/cid/ciy403 29771281 PMC6128403

[B27] Li X.-J. Zhou J. Liu L.-Q. Qian K. Wang C.-L. (2016). Identification of Genes in Longissimus Dorsi Muscle Differentially Expressed between Wannanhua and Yorkshire Pigs Using RNA-Sequencing. Anim. Genet. 47 (3), 324–333. 10.1111/age.12421 27038141

[B28] Lu F. Li J. Wang B. Cheng Y. Kong D.-H. Cui L. (2014). Profiling the Humoral Immune Responses to Plasmodium Vivax Infection and Identification of Candidate Immunogenic Rhoptry-Associated Membrane Antigen (RAMA). J. Proteomics 102, 66–82. 10.1016/j.jprot.2014.02.029 24607491

[B29] Lukacs-Kornek V. Malhotra D. Fletcher A. L. Acton S. E. Elpek K. G. Tayalia P. (2011). Regulated Release of Nitric Oxide by Nonhematopoietic Stroma Controls Expansion of the Activated T Cell Pool in Lymph Nodes. Nat. Immunol. 12 (11), 1096–1104. 10.1038/ni.2112 21926986 PMC3457791

[B30] Machado Siqueira A. Lopes Magalhães B. M. Cardoso Melo G. Ferrer M. Castillo P. Martin-Jaular L. (2012). Spleen Rupture in a Case of Untreated Plasmodium Vivax Infection. Plos Negl. Trop. Dis. 6 (12), e1934. 10.1371/journal.pntd.0001934 23272256 PMC3521714

[B31] Maier A. G. Rug M. O'Neill M. T. Brown M. Chakravorty S. Szestak T. (2008). Exported Proteins Required for Virulence and Rigidity of Plasmodium Falciparum-Infected Human Erythrocytes. Cell 134 (1), 48–61. 10.1016/j.cell.2008.04.051 18614010 PMC2568870

[B32] Marchesi S. Montani F. Deflorian G. D’Antuono R. Cuomo A. Bologna S. (2014). DEPDC1B Coordinates De-adhesion Events and Cell-Cycle Progression at Mitosis. Dev. Cell 31 (4), 420–433. 10.1016/j.devcel.2014.09.009 25458010 PMC4250264

[B33] Martin-Jaular L. Ferrer M. Calvo M. Rosanas-Urgell A. Kalko S. Graewe S. (2011). Strain-specific Spleen Remodelling in Plasmodium Yoelii Infections in Balb/c Mice Facilitates Adherence and Spleen Macrophage-Clearance Escape. Cell Microbiol. 13 (1), 109–122. 10.1111/j.1462-5822.2010.01523.x 20923452 PMC3228402

[B34] Miles A. Liaskou E. Eksteen B. Lalor P. F. Adams D. H. (2008). CCL25 and CCL28 Promote α4β7-integrin-dependent Adhesion of Lymphocytes to MAdCAM-1 under Shear Flow. Am. J. Physiology-Gastrointestinal Liver Physiol. 294 (5), G1257–G1267. 10.1152/ajpgi.00266.2007 18308860

[B35] Mitra S. K. Hanson D. A. Schlaepfer D. D. (2005). Focal Adhesion Kinase: in Command and Control of Cell Motility. Nat. Rev. Mol. Cell Biol. 6 (1), 56–68. 10.1038/nrm1549 15688067

[B36] Moog-Lutz C. Cavé-Riant F. Guibal F. C. Breau M. A. Di Gioia Y. Couraud P. O. (2003). JAML, a Novel Protein with Characteristics of a Junctional Adhesion Molecule, Is Induced during Differentiation of Myeloid Leukemia Cells. Blood 102 (9), 3371–3378. 10.1182/blood-2002-11-3462 12869515

[B37] Mueller S. C. Ghersi G. Akiyama S. K. Sang Q.-X. A. Howard L. Pineiro-Sanchez M. (1999). A Novel Protease-Docking Function of Integrin at Invadopodia. J. Biol. Chem. 274 (35), 24947–24952. 10.1074/jbc.274.35.24947 10455171

[B38] Mueller S. N. Matloubian M. Clemens D. M. Sharpe A. H. Freeman G. J. Gangappa S. (2007). Viral Targeting of Fibroblastic Reticular Cells Contributes to Immunosuppression and Persistence during Chronic Infection. Proc. Natl. Acad. Sci. 104 (39), 15430–15435. 10.1073/pnas.0702579104 17878315 PMC2000533

[B39] Nix D. A. Beckerle M. C. (1997). Nuclear-cytoplasmic Shuttling of the Focal Contact Protein, Zyxin: a Potential Mechanism for Communication between Sites of Cell Adhesion and the Nucleus. J. Cell Biol. 138 (5), 1139–1147. 10.1083/jcb.138.5.1139 9281590 PMC2136768

[B40] Ólafsson E. B. Ross E. C. Varas-Godoy M. Barragan A. (2019). Correction: TIMP-1 Promotes Hypermigration of Toxoplasma-Infected Primary Dendritic Cells via CD63-ITGB1-FAK Signaling. J. Cell Sci. 132 (5), 1. 10.1242/jcs.230920 30635444

[B41] Pardo A. Selman M. (2006). Matrix Metalloproteases in Aberrant Fibrotic Tissue Remodeling. Proc. Am. Thorac. Soc. 3 (4), 383–388. 10.1513/pats.200601-012TK 16738205

[B42] Prajapati S. K. Borlon C. Rovira-Vallbona E. Gruszczyk J. Menant S. Tham W.-H. (2019). Complement Receptor 1 Availability on Red Blood Cell Surface Modulates Plasmodium Vivax Invasion of Human Reticulocytes. Sci. Rep. 9 (1), 8943. 10.1038/s41598-019-45228-6 31221984 PMC6586822

[B43] Roa-Espitia A. L. Hernández-Rendón E. R. Baltiérrez-Hoyos R. Muñoz-Gotera R. J. Cote-Vélez A. Jiménez I. (2016). Focal Adhesion Kinase Is Required for Actin Polymerization and Remodeling of the Cytoskeleton during Sperm Capacitation. Biol. Open 5 (9), 1189–1199. 10.1242/bio.017558 27402964 PMC5051654

[B44] Roobsoong W. Tharinjaroen C. S. Rachaphaew N. Chobson P. Schofield L. Cui L. (2015). Improvement of Culture Conditions for Long-Term *In Vitro* Culture of Plasmodium Vivax. Malar. J. 14, 297. 10.1186/s12936-015-0815-z 26243280 PMC4524445

[B45] Rosen G. D. Dube D. S. (2006). ADHESION, CELL-MATRIX | Focal Contacts and Signaling. Encyclopedia Respir. Med. 1, 41–47. 10.1016/b0-12-370879-6/00013-2

[B46] Roy A. Ganesh G. Sippola H. Bolin S. Sawesi O. Dagälv A. (2014). Mast Cell Chymase Degrades the Alarmins Heat Shock Protein 70, Biglycan, HMGB1, and Interleukin-33 (IL-33) and Limits Danger-Induced Inflammation. J. Biol. Chem. 289 (1), 237–250. 10.1074/jbc.M112.435156 24257755 PMC3879547

[B47] Ryan D. Matthews J. (2005). Protein-protein Interactions in Human Disease. Curr. Opin. Struct. Biol. 15 (4), 441–446. 10.1016/j.sbi.2005.06.001 15993577

[B48] Schaller M. D. (2010). Cellular Functions of FAK Kinases: Insight into Molecular Mechanisms and Novel Functions. J. Cell Sci. 123 (Pt 7), 1007–1013. 10.1242/jcs.045112 20332118

[B49] Simeone P. Bologna G. Lanuti P. Pierdomenico L. Guagnano M. T. Pieragostino D. (2020). Extracellular Vesicles as Signaling Mediators and Disease Biomarkers across Biological Barriers. Ijms 21 (7), 2514. 10.3390/ijms21072514 PMC7178048 32260425

[B50] Sipilä K. Haag S. Denessiouk K. Käpylä J. Peters E. C. Denesyuk A. (2014). Citrullination of Collagen II Affects Integrin‐mediated Cell Adhesion in a Receptor‐specific Manner. FASEB J. 28 (8), 3758–3768. 10.1096/fj.13-247767 24823363

[B51] Svitkina T. (2018). The Actin Cytoskeleton and Actin-Based Motility. Cold Spring Harb Perspect. Biol. 10 (1), a018267. 10.1101/cshperspect.a018267 29295889 PMC5749151

[B52] Theocharis A. D. Skandalis S. S. Gialeli C. Karamanos N. K. (2016). Extracellular Matrix Structure. Adv. Drug Deliv. Rev. 97, 4–27. 10.1016/j.addr.2015.11.001 26562801

[B53] Toda H. Diaz-Varela M. Segui-Barber J. Roobsoong W. Baro B. Garcia-Silva S. (2020). Plasma-derived Extracellular Vesicles from Plasmodium Vivax Patients Signal Spleen Fibroblasts via NF-kB Facilitating Parasite Cytoadherence. Nat. Commun. 11 (1), 2761. 10.1038/s41467-020-16337-y 32487994 PMC7265481

[B54] Turner L. Lavstsen T. Berger S. S. Wang C. W. Petersen J. E. V. Avril M. (2013). Severe Malaria Is Associated with Parasite Binding to Endothelial Protein C Receptor. Nature 498 (7455), 502–505. 10.1038/nature12216 23739325 PMC3870021

[B55] Varo R. Chaccour C. Bassat Q. (2020). Update on Malaria. Medicina Clínica 155, 395–402. 10.1016/j.medcli.2020.05.010 32620355

[B56] Wang J. Dean D. C. Hornicek F. J. Shi H. Duan Z. (2019). RNA Sequencing (RNA-Seq) and its Application in Ovarian Cancer. Gynecol. Oncol. 152 (1), 194–201. 10.1016/j.ygyno.2018.10.002 30297273

[B57] Wang Z. Gerstein M. Snyder M. (2009). RNA-seq: a Revolutionary Tool for Transcriptomics. Nat. Rev. Genet. 10 (1), 57–63. 10.1038/nrg2484 19015660 PMC2949280

[B58] Weiss L. (1991). Barrier Cells in the Spleen. Immunol. Today 12 (1), 24–29. 10.1016/0167-5699(91)90108-6 2015045

[B59] Weiss L. (1989). Mechanisms of Splenic Control of Murine Malaria: Cellular Reactions of the Spleen in Lethal (Strain 17XL) Plasmodium Yoelii Malaria in BALB/c Mice, and the Consequences of Pre-infective Splenectomy. Am. J. Trop. Med. Hyg. 41 (2), 144–160. 10.4269/ajtmh.1989.41.144 2476037

[B60] World Health Organization (2020). World Malaria Report. Available at: https://www.who.int/teams/global-malaria-programme/reports/world-malaria-report-2020 (Accessed January 10, 2021).

[B61] Wu F. Chen L. Liu X. Su P. Li M. Yu X. (2010). A Novel CD29-like Protein Expressed in Japanese Lamprey (Lethenteron Japonicum) and Involved in Immune Response. Fish Shellfish Immunol. 29 (3), 407–413. 10.1016/j.fsi.2010.04.019 20488245

[B62] Yam X. Y. Niang M. Madnani K. G. Preiser P. R. (2017). Three Is a Crowd - New Insights into Rosetting in Plasmodium Falciparum. Trends Parasitol. 33 (4), 309–320. 10.1016/j.pt.2016.12.012 28109696

[B63] Yi M. Jiao D. Xu H. Liu Q. Zhao W. Han X. (2018). Biomarkers for Predicting Efficacy of PD-1/pd-L1 Inhibitors. Mol. Cancer 17 (1), 129. 10.1186/s12943-018-0864-3 30139382 PMC6107958

[B64] Zhao X.-K. Cheng Y. Liang Cheng M. Yu L. Mu M. Li H. (2016). Focal Adhesion Kinase Regulates Fibroblast Migration via Integrin Beta-1 and Plays a Central Role in Fibrosis. Sci. Rep. 6, 19276. 10.1038/srep19276 26763945 PMC4725867

