# Supplementary figures and images for: Identification of the Recombinant Plasmodium vivax Surface-Related Antigen as a Possible Immune Evasion Factor Against Human Splenic Fibroblasts by Targeting ITGB1
Source: Front Cell Dev Biol. 2021 Dec 6;9:764109. doi: 10.3389/fcell.2021.764109 (PMC8685506; doi:10.3389/fcell.2021.764109)

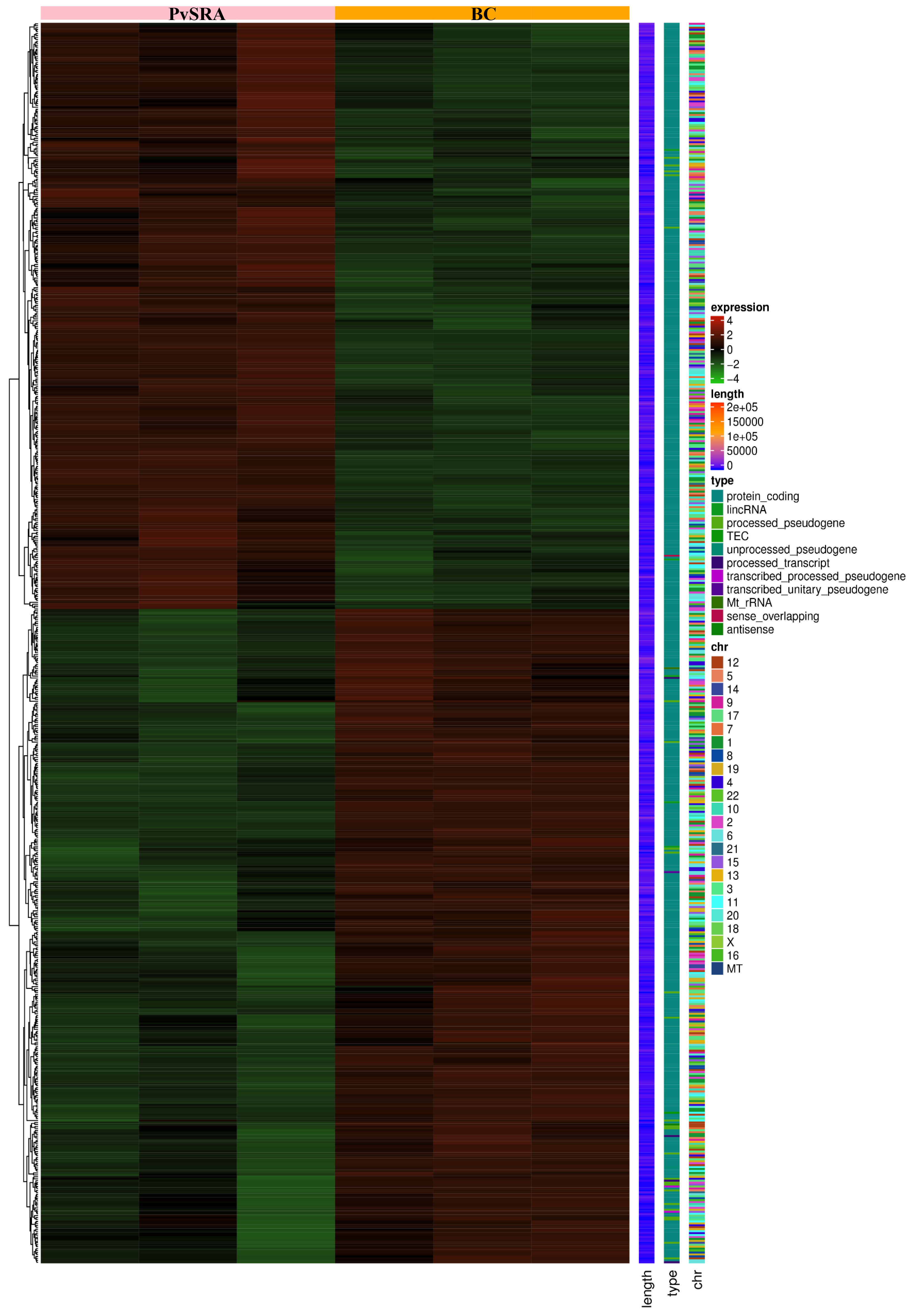

Supplement: Supplementary file 2 [file Image3.JPEG]

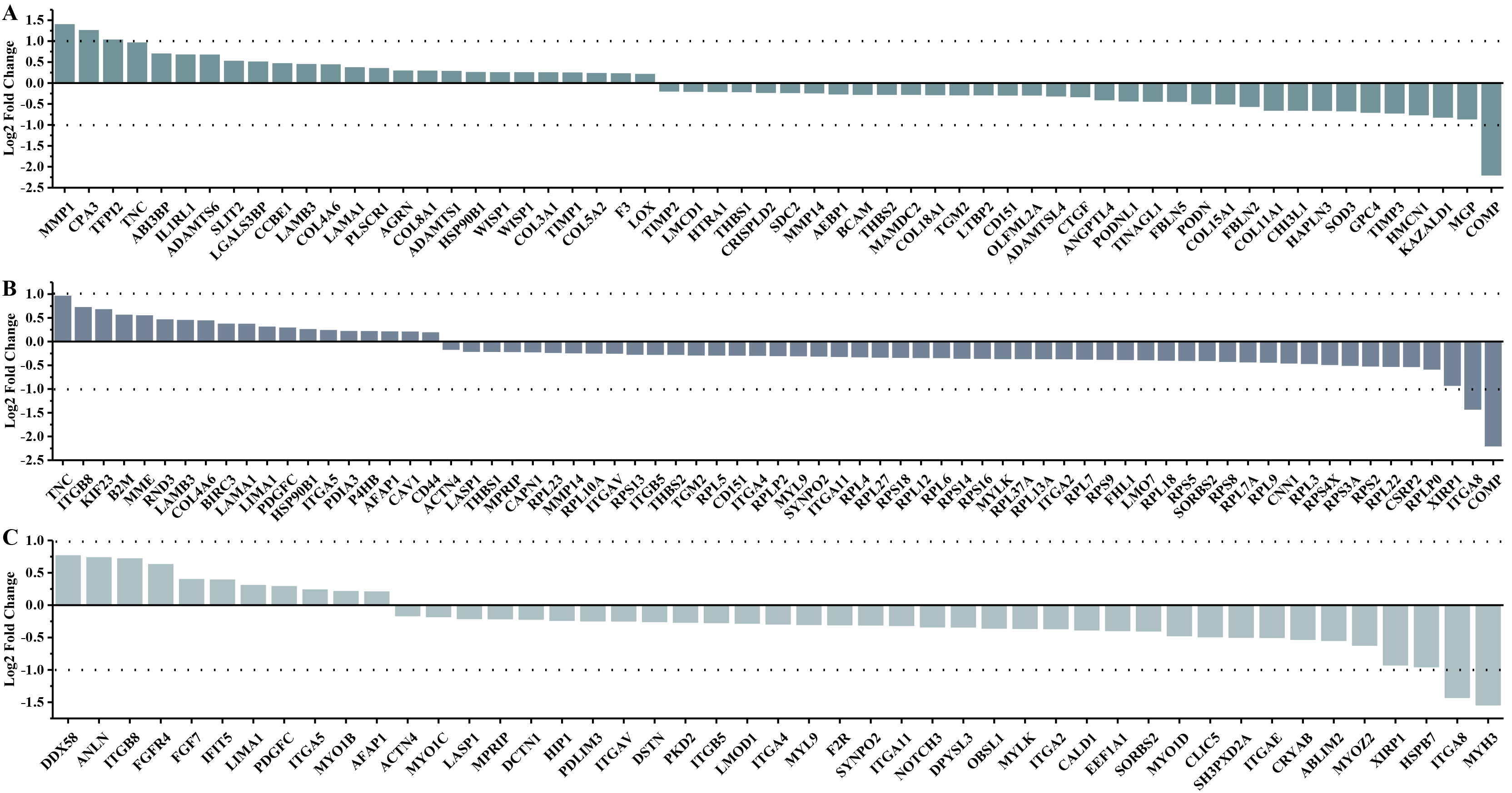

Supplement: Supplementary file 3 [file Image4.JPEG]

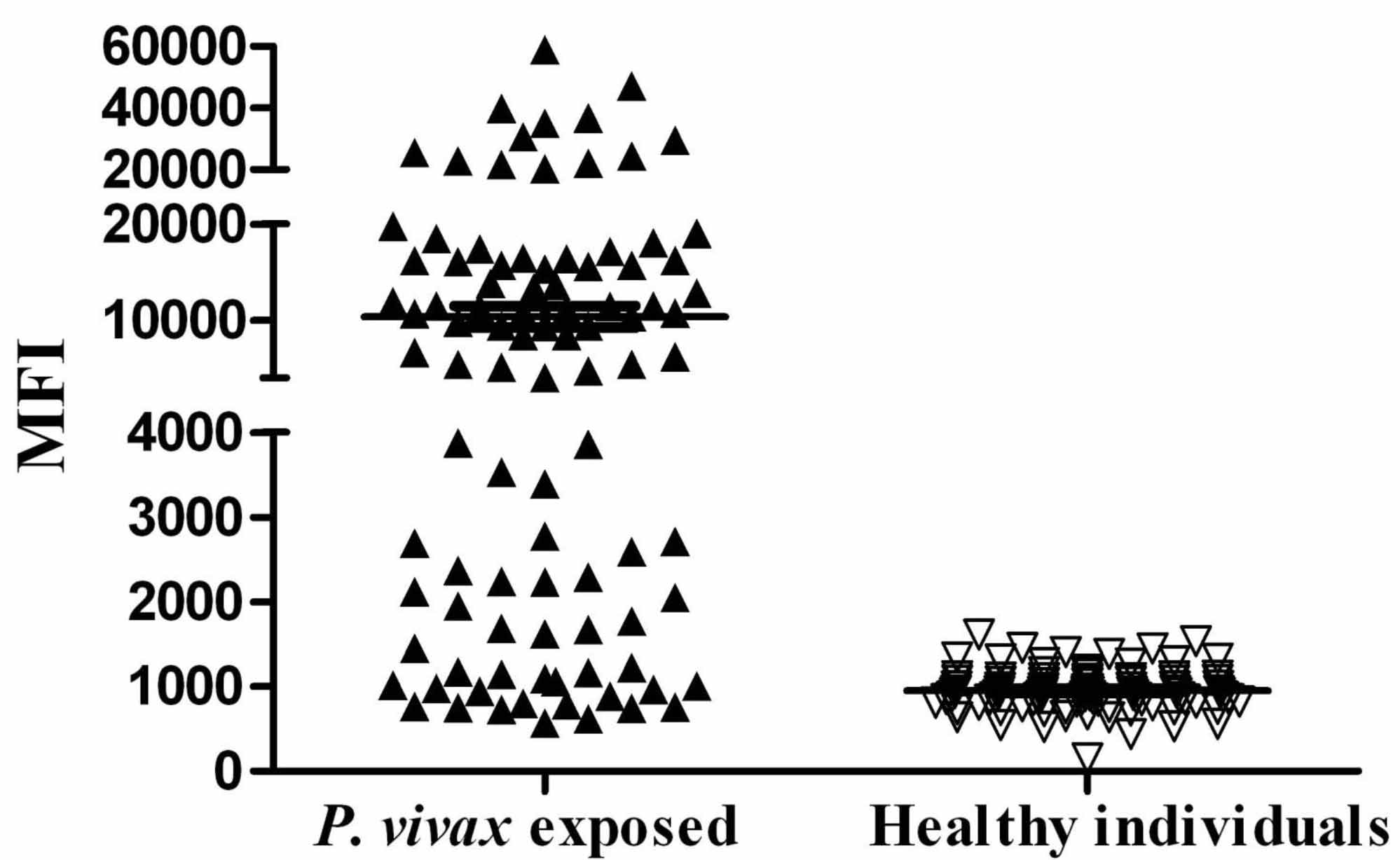

Supplement: Supplementary file 4 [file Image2.JPEG]

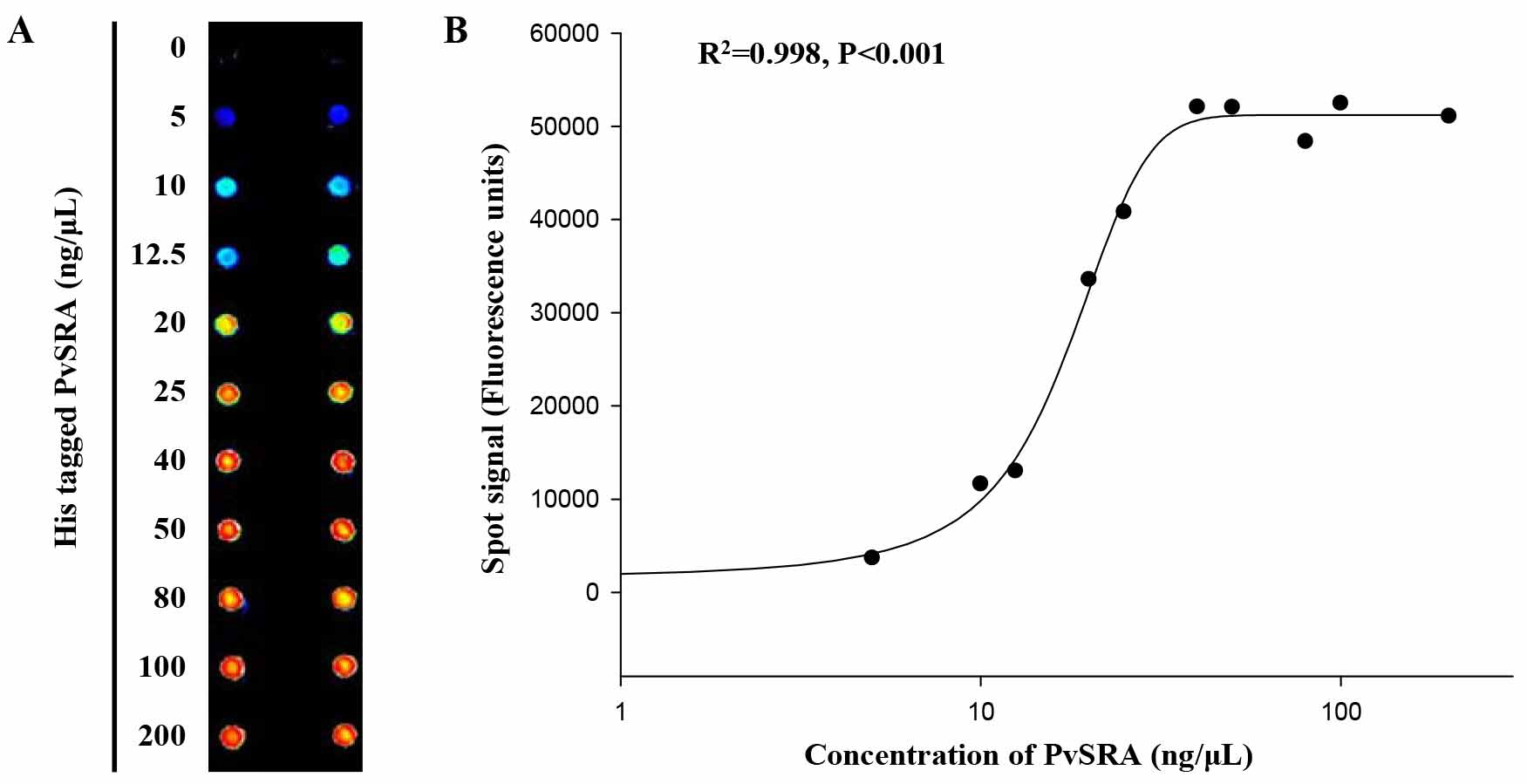

Supplement: Supplementary file 6 [file Image1.jpg]
